# Interfacial Engineering to Fabricate Nanoporous FeMo Bimetallic Nitride for Enhanced Electrochemical Ammonia Synthesis

**DOI:** 10.1002/advs.202410805

**Published:** 2024-12-18

**Authors:** Bin Fang, Liyuan Zhao, Yanqin Li, Nianliang Yin, Xin Wang, Jutao Jin, Wenlong Wang

**Affiliations:** ^1^ School of Materials Science and Engineering Dongguan University of Technology Dongguan Guangdong 523808 P. R. China; ^2^ School of Chemistry and Materials Science University of Science and Technology of China Hefei Anhui 230026 P. R. China; ^3^ School of Chemical Engineering and Energy Technology Dongguan University of Technology Dongguan Guangdong 523808 P. R. China; ^4^ Shenzhen Institute of Advanced Technology Chinese Academy of Sciences Shenzhen 518055 P. R. China

**Keywords:** bimetallic nitride, electrocatalysis, electrochemical ammonia synthesis, interface engineering

## Abstract

The electrochemical N_2_ reduction reaction (NRR) currently represents a green and sustainable approach to ammonia production. However, the further progress of NRR is significantly hampered by poor catalytic activity and selectivity, necessitating the development of efficient and stable electrocatalysts. Herein, a nanoporous Fe–Mo bimetallic nitride (Fe_3_N‐MoN) is synthesized using a molten‐salt preparation method. This catalyst demonstrates notable NRR performance, achieving a high NH_3_ yield rate of 45.1 µg h^−1^ mg^−1^ and a Faradaic efficiency (FE) of 26.5% at −0.2 V (vs RHE) under ambient conditions. Detailed experimental studies and density functional theory (DFT) calculations reveal that the fabricated interface between Fe_3_N and MoN effectively modulates the surface electronic structure of the catalyst. The interface induces an increase in the degree of electron deficiency at the nitrogen‐vacancy sites on the catalyst surface, allowing N_2_ molecules to occupy the nitrogen vacancies more easily, thereby promoting N_2_ adsorption/activation during the NRR process. Consequently, the Fe_3_N‐MoN catalyst exhibits outstanding NRR activity. The insights gained from fabricating the Fe_3_N‐MoN interface in this work pave the way for further development of interfacial engineering to prepare high‐efficient electrocatalyst.

## Graphical abstract

A nanoporous Fe‐Mo bimetallic nitride (Fe_3_N‐MoN) is successfully synthesized using a molten‐salt preparation method. This catalyst demonstrates notable NRR performance, achieving a high NH_3_ yield rate of 45.1 µg h^−1^ mg^−1^ and a Faradaic efficiency (FE) of 26.5% at −0.2 V (vs RHE) under ambient conditions, which surpasses the performance of both bare Fe_3_N and MoN significantly.

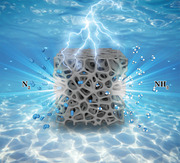

## 1 Introduction

As a crucial fundamental chemical in agriculture and industry, ammonia (NH_3_) holds ever‐growing significance for human society.^[^ ^1^, ^2^, ^3^ ^]^ However, to date, the predominant method of producing NH_3_ still relies heavily on the energy‐intensive Haber‐Bosch process, which operates under harsh conditions of 400–500 °C and 20–30 MPa.^[^ ^4^, ^5^, ^6^, ^7^ ^]^ In light of this situation, it is imperative to devise a novel process for the NH_3_ production route that is less polluting, consumes less energy, and is environmentally friendly.

Nowadays, the electrochemical NRR process for NH_3_ production has received widespread attention and rapid development, since this technology can utilize the electricity generated by renewable energy to convert inexhaustible small molecules of N_2_ and H_2_O into NH_3_ in electrolysis.^[^ ^8^, ^9^, ^10^, ^11^ ^]^ However, despite the promising potential of electrochemical NRR for NH_3_, the high chemical stability of nitrogen molecule (941 KJ mol^−1^) and the inevitable competition from the hydrogen evolution reaction (HER) in an aqueous environment pose significant challenges, resulting in relatively low NH_3_ yield and Faradaic efficiency.^[^ ^12^, ^13^, ^14^, ^15^ ^]^ Certainly, the development of efficient and robust electrocatalysts that significantly enhance the adsorption capacity of N_2_ molecules onto the catalyst surface in an aqueous medium, and facilitate the activation of adsorbed N_2_, holds paramount importance for the large‐scale production of NH_3_ via NRR technology.

Drawing inspiration from the fact that the principal active centers of nitrogenase for biological nitrogen fixation in nature are Fe and Mo elements, researchers have exerted significant efforts toward synthesizing a diverse array of Fe‐based and Mo‐based NRR catalysts, encompassing compounds such as FeS_2_,^[^ ^16^ ^]^ MoS_2_,^[^ ^17^ ^]^ Fe_2_O_3_,^[^ ^18^ ^]^ and MoO_2_.^[^ ^19^ ^]^ Recent research suggests that transition metal nitrides (TMNs) exhibit significant potential for NRR under ambient conditions, attributed to their exceptional electronic conductivity and robust chemical stability. Furthermore, owing to the presence of N atoms within the bulk phase, the reaction path of TMNs catalysts during NRR favors the efficient Mars‐van Krevelen (MvK) mechanism, thereby endowing TMNs with an inherent advantage compared to other compounds.^[^ ^20^, ^21^ ^]^ Currently, the utilization of MoN materials in NRR has garnered significant attention and research reports. Nonetheless, as of our knowledge, the exploration of Fe_x_N materials as catalysts for NRR is rather limited, with scare documentation available. Moving a step forward, there is a distinct absence of reports on the synthesis of bimetallic nitrides comprising both Fe and Mo and their potential application in NRR.

Interface engineering, a pivotal aspect in catalyst design, involves the intricate interplay between various components within a catalyst, offering novel approaches to elevate catalytic performance. The interface within a catalyst serves as the boundary between two distinct components, facilitating the equilibrium of adsorption and desorption of reactants and intermediates during catalysis, accelerating electron transfer, and playing a pivotal role in enhancing electrochemical performance. Consequently, a series of efficient catalysts have been recently designed by researchers through the rational construction of interface engineering strategies, and these catalysts have been subsequently applied to various electrocatalytic reactions, including water splitting,^[^ ^22^, ^23^ ^]^ NRR^[^ ^24^, ^25^ ^]^ and CO_2_ reduction reaction (CO_2_RR).^[^ ^26^, ^27^ ^]^ Correspondingly, the development of a crystal interface through the fabrication of a bimetallic nitride holds immense potential in promoting NRR activity, as it possesses the ability to modulate the surface electronic structure of the catalyst, expedite electron transport, and enhance the adsorption capacity of N_2_. Nevertheless, the pertinent research in this area remains unexplored.

In this contribution, the nanoporous Fe‐Mo bimetallic nitride catalyst (Fe_3_N‐MoN) has been successfully prepared through a molten salt approach, exhibiting remarkable NRR performance. Specifically, the Fe_3_N‐MoN catalyst exhibits a high NH_3_ yield rate of 45.1 µg h^−1^ mg^−1^ and a notable Faradaic efficiency (FE) of 26.5% at −0.2 V (vs RHE) under ambient conditions, surpassing the performance of both bare Fe_3_N and MoN significantly. Density functional theory (DFT) calculations were utilized to elucidate the synergistic effect of Fe_3_N and MoN. The findings indicate that the constructed interface between these two substances plays a pivotal role in enhancing NRR activity. Specifically, this interface aids in expanding electron‐deficient regions at the nitrogen‐vacancy sites, thereby improving the adsorption capacity of N_2_ to the nitrogen vacancies in NRR, ultimately resulting in favorable NRR performance.

## 2 Results and Discussion

### 2.1 Synthesis and Characterization of Fe_3_N‐MoN

**Figure 1:**
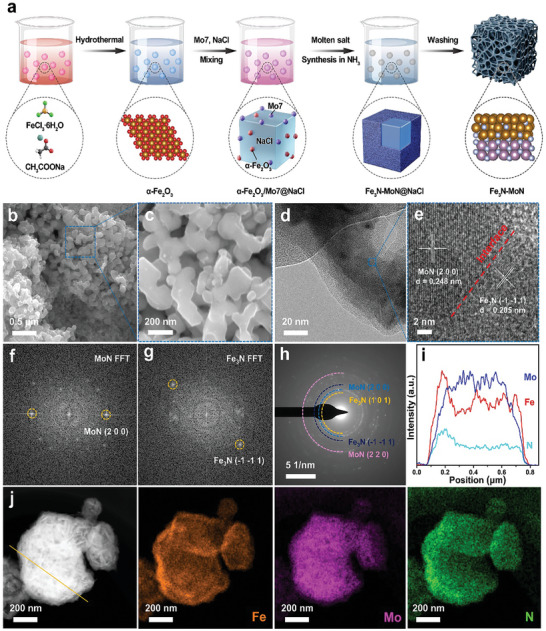
a) Schematic illustration of the molten salt synthesis route of Fe_3_N‐MoN. b,c) SEM images of Fe_3_N‐MoN. d) TEM image of Fe_3_N‐MoN. e) HRTEM image of Fe_3_N‐MoN. f,g) The selected area FFT patterns from image e. h) SAED image of Fe_3_N‐MoN. i) STEM‐EDX line profile of Fe_3_N‐MoN from the region depicted in j j) HAADF‐STEM images accompanied by corresponding element mapping analyses of Fe_3_N‐MoN.

The schematic illustration of the fabrication process of Fe_3_N‐MoN is presented in **Figure** **1**a. The synthesis of Fe_3_N‐MoN involves four steps. First, the α‐Fe_2_O_3_ was prepared through a one‐step hydrothermal method. Second, the synthesized α‐Fe_2_O_3_, ammonium molybdate (Mo7), and NaCl are uniformly and thoroughly mixed in ethanol to obtain α‐Fe_2_O_3_/Mo7@NaCl mixture. Third, the above mixture of α‐Fe_2_O_3_/Mo7@NaCl was subjected to a nitridation treatment in an NH_3_ atmosphere, resulting in the product of Fe_3_N‐MoN@NaCl. Finally, Fe_3_N‐MoN@NaCl was subjected to repeated washing in deionized water, thereby removing the NaCl salt templates, and the resulting product was denoted as Fe_3_N‐MoN. The morphology and surface structure of Fe_3_N‐MoN were thoroughly examined using a range of characterization techniques, including scanning electron microscope (SEM), transmission electron microscopy (TEM), selected area electron diffraction (SAED), and high‐angle annular dark‐field scanning transmission electron microscopy (HAADF‐STEM). Additionally, X‐ray photoelectron spectroscopy (XPS) and X‐ray absorption near‐edge structure (XANES) spectra were employed to further analyze the chemical composition and electronic structure of the catalyst. The SEM results depicted in Figure 1b,c reveal that Fe_3_N‐MoN exhibits a uniform porous distribution structure, accompanied by splendid spatial connectivity. This distinctive structure significantly contributes to the rapid and stable adsorption of N_2_. The HRTEM images (Figure 1d,e) indicate that Fe_3_N‐MoN possesses excellent crystallinity. Specifically, two distinct interplanar spacings of 0.205 and 0.248 nm can be attributed to the Fe_3_N (−1 −1 1) and MoN (2 0 0) crystal plane, respectively. In addition, HRTEM images of a large field of view containing the two components is shown in Figure S1 (Supporting Information). Notably, the interface between these two components is clearly observable, indicating a well‐defined interface structure. These findings were further corroborated by the selective area Fast‐Fourier‐transform (FFT) patterns depicted in Figure 1f,g. The diffraction rings evident in the SAED pattern explicitly demonstrate the polycrystallinity nature of Fe_3_N‐MoN, as illustrated in Figure 1h. Furthermore, the STEM‐EDX line scan (Figure 1i) and HAADF‐STEM images (Figure 1j) offer insights into the uniform distribution of Fe, Mo, and N elements within the Fe_3_N‐MoN composite. As part of the control experiments, the Fe_3_N and MoN catalysts were synthesized and characterized, as presented in Figures S2–S5 (Supporting Information). Additionally, Figure S6 (Supporting Information) showcases the nitrogen adsorption–desorption isotherms of these samples, revealing their respective specific surface areas: Fe_3_N‐MoN (25.97 m^2^ g^−1^), Fe3N (19.51 m^2^ g^−1^) and MoN (14.08 m^2^ g^−1^), and this comparison clearly demonstrates that the interface fabrication enhances the specific surface area of the material.

**Figure 2:**
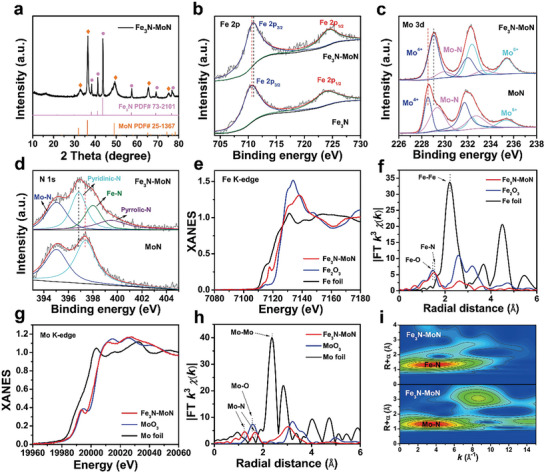
a) XRD pattern of Fe_3_N‐MoN. b) XPS spectra of Fe 2p, c) XPS spectra of Mo 3d, d) N 1s XPS spectra of Fe_3_N‐MoN. e) Fe K‐edge XANES spectra, f) Fe K‐edge FT ‐EXAFS spectra, g) Mo K‐edge XANES spectra, h) Mo K‐edge FT‐EXAFS spectra of Fe_3_N‐MoN and reference samples. i) WT‐EXAFS of Fe_3_N‐MoN.

As depicted in **Figure** **2**a, a comprehensive examination of the crystal structure of Fe_3_N‐MoN was conducted through Powder X‐ray diffraction (XRD) analysis. The Fe_3_N‐MoN catalyst exhibited distinct diffraction peaks that are attributable to Fe_3_N (PDF# 73–2101) and MoN (PDF# 25–1367), indicating the successful fabrication of the interface and preservation of their respective crystal structures. We further implemented Raman analysis to elaborate the surface state of Fe_3_N‐MoN, as shown in Figure S7 (Supporting Information), the resulting peaks correspond well to the characteristic peaks of Fe_3_N and MoN, suggesting successful hybridization. The surface composition and chemical state of the hetero‐interface Fe_3_N‐MoN were further analyzed using X‐ray photoelectron spectroscopy (XPS). As presented in Figure S8 (Supporting Information), the XPS survey spectra reveal the existence of Fe, Mo, and N elements, which is consistent with the results obtained from HAADF‐STEM. As depicted in Figure 2b, the high‐resolution Fe 2p spectra of Fe_3_N‐MoN exhibit two prominent peaks, centered at 710.95 and 724.56 eV, respectively. These peaks can be ascribed to Fe 2p_3/2_ and Fe 2p_1/2_ spin‐orbit states, consistent with previous studies.^[^ ^28^, ^29^ ^]^ The high‐resolution Mo 3d spectra, shown in Figure 2c, can be deconvoluted into six peaks. The peaks positioned at 229.03/232.02 eV and 232.38/235.38 eV correspond to the Mo^4+^ and Mo^6+^ oxidation states, respectively. Furthermore, the peaks situated at 229.95/233.46 eV could be assigned to Mo─N bonding present in MoN, in line with previous reports.^[^ ^30^, ^31^ ^]^ In the high‐resolution N 1s spectra (Figure 2d), distinct peaks are observed at 396.84, 399.56, 395.0, and 397.98 eV. These peaks correspond to pyridinic N, pyrrolic N, molybdenum coordination N (Mo─N), and iron coordination N (Fe─N), respectively, aligning with previous findings.^[^ ^32^ ^]^ Notably, when compared to the pristine Fe_3_N and MoN catalyst, the Fe 2p and Mo 3d spectra of Fe_3_N‐MoN exhibit a noticeable shift toward higher binding energies. Concurrently, the N 1s spectrum shows a shift toward lower binding energies. This result clearly indicates the occurrence of significant electron transfer between the two components of the heterojunction.

To further explore the atomic‐level fine structure of the catalyst, the techniques of X‐ray Absorption Near‐edge Spectroscopy (XANES) and Extended X‐ray Absorption Fine Structure (EXAFS) within synchrotron radiation technology are employed to analyze the coordination environment and chemical valence of Fe and Mo atoms in Fe_3_N‐MoN. Figure 2e portrays Fe K‐edge profiles of Fe_3_N‐MoN, Fe_2_O_3,_ and Fe foil, revealing that the average valence of Fe atom lies between Fe^0^ and Fe^3+^, aligning with the XPS results of XPS depicted in Figure 2b.^[^ ^33^ ^]^ The Fe K‐edge Fourier transformed EXAFS (FT‐EXAFS) spectrum of Fe_3_N‐MoN (Figure 2f) exhibits a distinct peak at 1.5 Å, attributed to Fe‐N bonding.^[^ ^34^, ^35^ ^]^ Figure 2g depicts the Mo K‐edge profiles of Fe_3_N‐MoN, MoO_3_, and Mo foil, indicating that the average valence of Mo atoms falls between Mo^0^ and Mo^6+^.^[^ ^36^, ^37^ ^]^ Furthermore, the Mo K‐edge FT‐EXAFS spectrum of Fe_3_N‐MoN exhibits a clear peak at 1.25 Å, which could be ascribed to Mo─N bonding.^[^ ^38^ ^]^ The wavelet transforms (WT) of Fe_3_N‐MoN presented in Figure 2i further validate the formation of Fe‐N and Mo─N bonding in Fe_3_N‐MoN.

### 2.2 NRR Performance of Fe_3_N‐MoN

**Figure 3:**
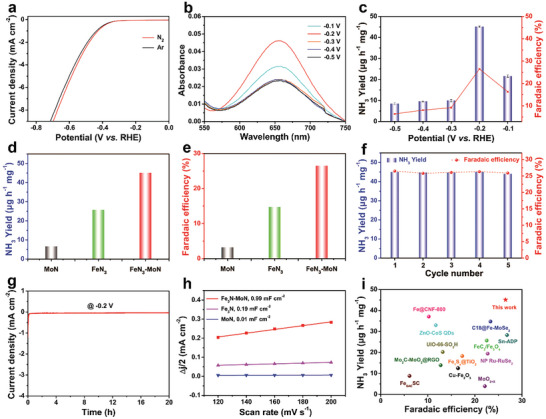
a) LSV curves of Fe_3_N‐MoN in 0.1 m HCl saturated with Ar and N_2_. b) UV–vis absorption spectra of Fe_3_N‐MoN under different applied potentials. c) NH_3_ yields and FEs of Fe_3_N‐MoN under different applied potentials. d) Comparative analysis of NH_3_ yields between Fe_3_N‐MoN, Fe_3_N, and MoN. e) FE comparison of Fe_3_N‐MoN, Fe_3_N, and MoN. f) Cycling stability tests of Fe_3_N‐MoN at −0.2 V (vs RHE). g) Chronoamperometry curve of Fe_3_N‐MoN over a duration of 20 h. h) Capacitive current densities at 0.15 V (versus RHE) as a function of scan rates for Fe_3_N‐MoN, Fe_3_N, and MoN. i) A comparative evaluation of the NRR performance of Fe_3_N‐MoN with recently reported electrocatalysts.

The electrocatalytic NRR performance of the synthesized Fe_3_N‐MoN catalyst is rigorously evaluated in 0.1 m HCl electrolyte in an H‐type cell partitioned by a Nafion 117 membrane, under ambient conditions. For the experiment, carbon papers loaded with the prepared catalysts serve as the working electrode, while Pt foil functions as the counter electrode, and an Ag/AgCl immersed in saturated KCl electrolyte acts as the reference electrode. The indophenol blue method^[^ ^39^ ^]^ and Watt–Chrisp approach^[^ ^40^ ^]^ are employed for quantitative detection of the generated NH_3_ product and the possible by‐product N_2_H_4_, with the respective calibration curves depicted in Figures S9 and S10 (Supporting Information). Initially, the linear sweep voltammetry (LSV) curves of Fe_3_N‐MoN were measured under varying conditions, as depicted in **Figure** **3**a. A conspicuous observation was that the current density in N_2_‐saturated electrolyte was significantly higher compared to that in Ar‐saturated electrolyte, indicating the occurrence of NRR. Subsequently, the NRR activity was further investigated by chronoamperometry tests conducted at varying potentials ranging from −0.1 to −0.5 V (vs RHE) in an N_2_‐saturated electrolyte. Based on the UV–vis absorption spectra of the electrolytes (Figure 3b), the NH_3_ yield rate and FE of Fe_3_N‐MoN were calculated through indophenol blue method, as illustrated in Figure 3c. It is evident that at −0.2 V (vs RHE), the highest NH_3_ yield and FE were obtained, reaching 45.1 µg h^−1^ mg^−1^ and 26.5% respectively. The NRR performance of both bare Fe_3_N and MoN samples was also scrutinized under identical testing conditions, as depicted in Figures S11 and S12 (Supporting Information). Upon comparing their performance at the optimal voltage of −0.2 V (vs RHE) (Figure 3d,e), it becomes evident that both Fe_3_N and MoN exhibit significantly inferior NH_3_ yield and FE compared to the Fe_3_N‐MoN composite. These findings underscore the pivotal role played by the synthesized bimetallic nitrides and the heterojunction structures between them in the reduction of N_2_ to NH_3_. To validate that the NH_3_ originated from the NRR process catalyzed by Fe_3_N‐MoN and conformed to the Mars‐van Krevelen (MvK) mechanism, comparative experiments were conducted. These experiments involved using carbon paper as the working electrode in N_2_ gas at −0.2 V for 1 h and using Fe_3_N‐MoN as the working electrode in both N_2_ and Ar gas at −0.2 V for 1 h. As depicted in Figure S13 (Supporting Information), based on the analyzed UV–vis absorption spectra and NRR activities, it is evident that the electrolyte and carbon paper (CP) do not contribute to the production of nitrogen‐containing compounds. Furthermore, the detection of NH_3_ through electrolysis in an Ar atmosphere is attributed to the protonation of N atoms within the Fe_3_N‐MoN phase, aligning with the MvK mechanism. Then we conducted ^15^N isotopic labeling experiment to further confirm the NH_3_ is derived from NRR and through the MvK mechanism. As shown in Figure S14 (Supporting Information), when ^14^N_2_ was used as feed gas in the NRR process, the ^1^H nuclear magnetic resonance (NMR) spectra exhibits triplet peaks, which is in line with ^1^H NMR spectrum of ^14^NH_4_^+^ standard. When ^15^N_2_ was used as feed gas in the NRR process, the triplet peaks of ^14^NH_4_^+^ and doublet peaks of ^15^NH_4_^+^ appear simultaneously. And the content of the triplet peaks only accounts for a small part, demonstrating that a small part of the NH_3_ produced comes from the hydrogenation of N atoms of the Fe_3_N‐MoN itself, and most of NH_3_ comes from the electrochemical conversion of ^15^N_2_. These results strongly verify that the detected NH_3_ originates from the NRR reaction and follows the MvK mechanism. Additionally, the potential production of N_2_H_4_ during the NRR process catalyzed by Fe_3_N‐MoN through Watt–Chrisp approach was also investigated. As illustrated in Figure S15 (Supporting Information), the negligible amount of N_2_H_4_ demonstrates outstanding selectivity of the Fe_3_N‐MoN catalyst.

In practical applications, the stability and recyclability of catalysts are paramount. Therefore, as depicted in Figure 3f, the Fe_3_N‐MoN catalyst was subjected to five consecutive cycling tests at the optimal voltage of −0.2 V (vs RHE). It is evident that after the tests, there was no apparent change in NH_3_ yield and FE. Subsequently, it was found that the Fe_3_N‐MoN catalyst exhibited negligible changes in its current density during the consecutive chronoamperometry measurement at −0.2 V (vs RHE) for a duration of 20 h (Figure 3g). These results indicate that the Fe_3_N‐MoN material exhibits exceptional stability as a NRR catalyst. Moreover, the SEM image of Fe_3_N‐MoN for duration of 20 h (Figure S16, Supporting Information) shows that the nanoporous structure is well preserved after a long‐term electrolysis. This result indicates that Fe_3_N‐MoN possesses excellent structural stability. To further elucidate the underlying cause of the high catalytic activity of Fe_3_N‐MoN, the electrochemical surface area (ECSA) was evaluated. Based on the positive correlation between ECSA and the double‐layer capacitance (*C*_dl_), the *C*_dl_ values of catalysts were calculated using cyclic voltammetry (CV) measurements (Figure S17, Supporting Information). As depicted in Figure 3h, the Fe_3_N‐MoN catalyst exhibits the highest *C*_dl_ value of 0.99 mF cm^−2^, surpassing Fe_3_N (0.19 mF cm^−2^) and MoN (0.01 mF cm^−2^), indicating that the Fe_3_N‐MoN catalyst exposes the greatest number of electrochemical active sites. This finding aligns well with the BET results presented in Figure S6 (Supporting Information). Ultimately, when compared with recent state‐of‐art NRR electrocatalysts, the Fe_3_N‐MoN emerges as a dominant catalyst, excelling in both NH_3_ yield and FE (Figure 3i).^[^ ^19^, ^24^, ^41^, ^42^, ^43^, ^44^, ^45^ ^–^ ^50^ ^]^

### 2.3 Proposed Mechanism

**Figure 4:**
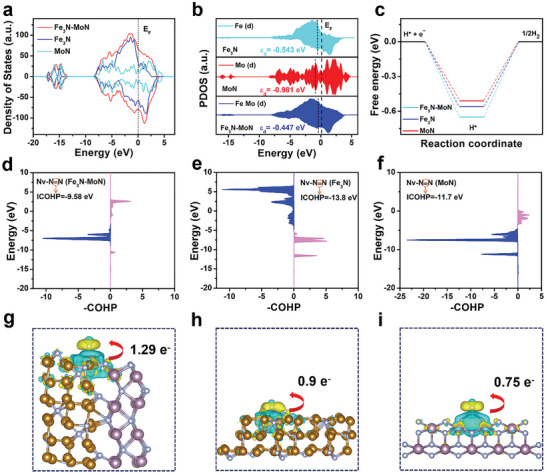
a) Density of states calculations for Fe_3_N‐MoN, Fe_3_N, and MoN. b) Visualization of d‐band center for Fe_3_N‐MoN, Fe_3_N, and MoN. c) Calculated free energy diagrams of HER on Fe_3_N‐MoN, Fe_3_N, and MoN surfaces. d,e,f) Crystal orbital Hamilton population (COHP) analysis between the N─N bonds of the N_2_ adsorbed at the Nv site for Fe_3_N‐MoN, Fe_3_N, and MoN, respectively. g,h,i) Charge density differences and Bader analysis of the N_2_ adsorbed at the Nv site for Fe_3_N‐MoN, Fe_3_N, and MoN, respectively.

To gain a deeper understanding of the inherent catalytic activity of Mo_5_N_6_‐Ni_3_S_2_ HNPs/NF, we conducted density functional theory (DFT) calculations to analyze the Gibbs free energy of To gain a deeper understanding of how the heterojunction structure enhances NRR activity, DFT calculations were conducted. The simulation model for Fe_3_N‐MoN was constructed using Fe_3_N (−1 –1 1) and MoN (2 0 0) fragments. Initially, the density of states (DOS) for Fe_3_N‐MoN, Fe_3_N, and MoN were calculated, as depicted in **Figure** **4**a. It is evident that the electron density near the Fermi level of Fe_3_N‐MoN is higher than that of bare Fe_3_N and MoN, suggesting a more abundant distribution of electrons on the surface of Fe_3_N‐MoN. This augmentation in electron availability can potentially facilitate the activation of N_2_, thus contributing to its NRR activity. Second, the d band center was calculated for three models: Fe_3_N‐MoN (−0.447 eV), Fe_3_N (−0.543 eV), and MoN (−0.981 eV), as depicted in Figure 4b. When compared to Fe_3_N and MoN, the d band center of Fe_3_N‐MoN exhibits a shift toward the Fermi level, indicating a strengthening of the bonding interaction between the catalytic sites and intermediates. These findings underscore the significant role that the formation of the interface plays in effectively optimizing the electronic structure of the heterogeneous structure. Additionally, the evaluation of the competitive HER process has been conducted, as illustrated in Figure 4c and Figure S18 (Supporting Information), it is evident that the HER tends to occur more easily on MoN and Fe_3_N, which suggests that the Fe_3_N‐MoN composite effectively suppresses the HER process.

To further validate the reaction pathway of the catalysts adheres to the Mars‐van Krevelen (MvK) mechanism, the Gibbs free energy (ΔG) diagrams for NRR over Fe_3_N‐MoN via the distal pathway, alternating pathway and MvK pathway have been calculated. The process schematic diagrams representing these pathways are shown in Figure S19 (Supporting Information). The NRR reaction energy barrier depicted in Figure S20 (Supporting Information) reveals that the distal and alternating pathways possess a significantly higher rate‐determining step (RDS) of 1.01 eV, in contrast to the MvK pathway, which exhibits a lower RDS of 0.78 eV. This result validates the energetic preference of the MvK pathway for Fe_3_N‐MoN, indicating its superior thermodynamic favorability. In Figure S21 (Supporting Information), a pivotal observation is made regarding the MvK mechanism, specifically the adsorption of N_2_ that occurs in the sixth step, as N_2_ enters the nitrogen vacancy (Nv). To gain a deeper understanding of this process, we conducted a detailed analysis of the charge density difference in the formed Nv, as presented in Figure S21 (Supporting Information). Notably, all three structures display an electron‐deficient region at the Nv. However, a notable distinction arises when comparing Fe_3_N‐MoN with Fe_3_N and MoN, where the electron‐deficient area in Fe_3_N‐MoN is significantly more pronounced. This significant electron deficiency in Fe_3_N‐MoN enhances its capacity to readily accept the lone pair electrons of N_2_, thus facilitating the adsorption process. To further investigate the activation process of N_2_ upon its entry into the Nv, the bond strength of the N≡N triple bonds of the adsorbed N_2_ was calculated via the crystal orbital Hamilton population (COHP) analysis. As exhibited in Figure 4d–f, the integrated crystal orbital Hamilton population (ICOHP) values for the N≡N bonds in Fe_3_N‐MoN, Fe_3_N, and MoN are −9.58, −13.8 and −11.7 eV, respectively. Notably, the relatively positive ICOHP value observed for Fe_3_N‐MoN suggests that the N≡N bond adsorbed on Fe_3_N‐MoN exhibits greater instability and activation compared those on Fe_3_N and MoN. Furthermore, to visually examine the electron transfer between the adsorbed N_2_ and the catalyst, charge density differences and Bader charge analysis were conducted. As shown in Figure 4g–i, 1.29 e^−^ could be donated to the *N_2_ intermediate from the surface of Fe_3_N‐MoN, exceeding that observed for Fe_3_N (0.9 e^−^) and MoN (0.75 e^−^). These computational findings indicate that the formation of the heterojunction interface plays a pivotal role in enhancing the adsorption of N_2_ at the Nv sites and its subsequent activation.

**Figure 5:**
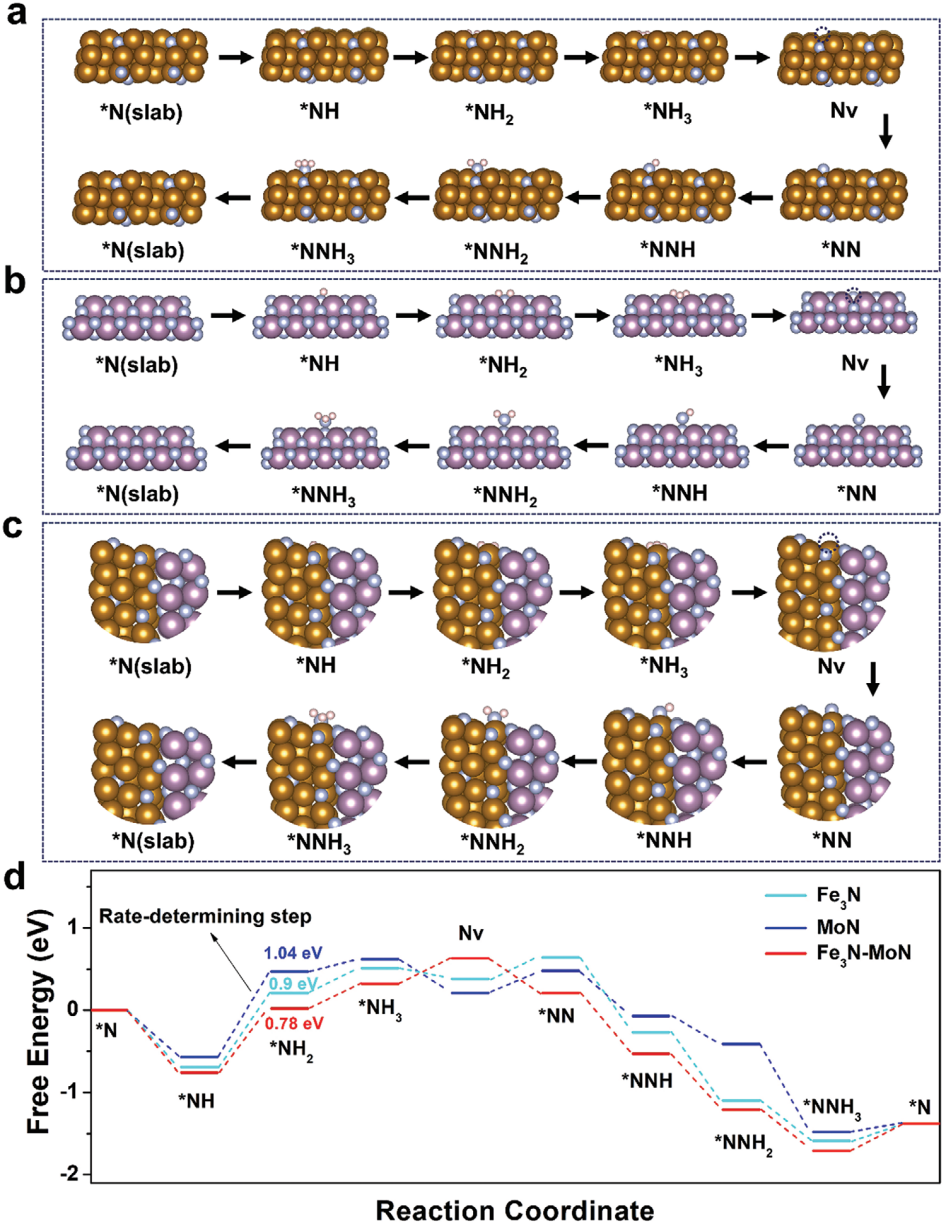
Schematic diagrams of the Mars‐van Krevelen (MvK) mechanism pathways for three different catalysts: a) Fe_3_N‐MoN, b) Fe_3_N, and c) MoN. d) Calculated Gibbs free energy diagrams of each step in MvK pathway for Fe_3_N‐MoN, Fe_3_N, and MoN.

Finally, the Gibbs free energy (*ΔG*) diagrams pertaining to NRR via MvK pathway for Fe_3_N‐MoN, Fe_3_N, and MoN were meticulously calculated, and their process schematic diagrams are presented in **Figure** **5**a–c. As illustrated in Figure 5d, it is evident that the rate‐determining step (RDS) for all three structures under investigations is the reductive protonation of adsorbed *NH, specifically the reaction *NH + H^+^ + e^−^ →*NH_2_. Notably, Fe_3_N‐MoN exhibits the lowest energy barrier among them, amounting to 0.78 eV. From a thermodynamical perspective, the reaction rate (k) can be quantified using the Arrhenius equation: *k* = Aexp(−*ΔE*/RT), where A represents the pre‐exponential constant, *ΔE* denotes the energy barrier, R stands for the gas constant, and T is the temperature. Given this relationship, k is directly influenced by *ΔE*, where a lower energy barrier translates to a higher reaction rate. Consequently, the constructed Fe_3_N‐MoN interface effectively lowers the energy barrier for the NRR, thereby theoretically enhancing the reaction rate.

## 3 Conclusion

In summary, a nanoporous Fe‐Mo bimetallic nitride catalyst (Fe_3_N‐MoN) has been successfully prepared using a molten‐salt synthesis approach. Owing to the unique properties of bimetallic nitrides, the as‐formed Fe_3_N‐MoN exhibited to possess superior NRR performance under the Mars‐van Krevelen (MvK) mechanism, achieving a high NH_3_ yield rate of 45.1 µg h^−1^ mg^−1^ and a FE of 26.5% at −0.2 V (vs RHE). Experimental studies and DFT calculations suggest that the formation of the heterojunction interface between Fe_3_N and MoN plays a vital role in tuning the electronic structure of the Fe_3_N‐MoN catalyst. Specifically, it is found that the interface enhances the degree of an electron‐deficient area at the nitrogen vacancies on the catalyst surface, making it more conducive for N_2_ to enter the nitrogen vacancies and facilitating the adsorption/activation of N_2_ during the NRR process. From a thermodynamic perspective, the Fe_3_N‐MoN catalyst is demonstrated to significantly reduce the reaction energy barrier for the reductive protonation of adsorbed *NH (*NH + H^+^ + e^−^ →*NH_2_), leading to an accelerated reaction rate and remarkable NRR performance. This work first introduced the application of bimetallic nitrides in the field of NRR, and analyzed the essential reason for the high performance of this catalyst from the perspective of catalyst structure and structure‐activity relationship. The Fe_3_N‐MoN catalyst, which does not contain precious metals, is a typical example of a high‐activity and cost‐effective NRR catalyst achieved through interface engineering, paving the way for further research in this field. The development of NRR catalyst with higher activity and stability is ongoing in our laboratory.

## Conflict of Interest

The authors declare no conflict of interest.

## Supporting information



Supporting Information

## Data Availability

The data that support the findings of this study are available from the corresponding author upon reasonable request.
