# Supplementary material for: Interfacial Engineering to Fabricate Nanoporous FeMo Bimetallic Nitride for Enhanced Electrochemical Ammonia Synthesis
Source: Adv Sci (Weinh). 2024 Dec 18;12(7):2410805. doi: 10.1002/advs.202410805 (PMC11831536; doi:10.1002/advs.202410805)
Supplement: Supplementary file 1 — Supporting Information [file ADVS-12-2410805-s001.docx]

Supporting Information

Interfacial Engineering to Fabricate Nanoporous FeMo Bimetallic Nitride for Enhanced Electrochemical Ammonia Synthesis

*Bin Fang, Liyuan Zhao, Yanqin Li, Nianliang Yin, Xin Wang*, Jutao Jin* and Wenlong Wang**

B. Fang, Y. Li, N. Yin, J. Jin, Prof. W. Wang

School of Materials Science and Engineering

Dongguan University of Technology

Dongguan, Guangdong, 523808, People's Republic of China

E-mail: wangwl@dgut.edu.cn, jinjt@dgut.edu.cn

L. Zhao

School of Chemical Engineering and Energy Technology

Dongguan University of Technology

Dongguan, Guangdong, 523808, People's Republic of China

X. Wang

Shenzhen Institute of Advanced Technology

Chinese Academy of Sciences

Shenzhen 518055, People's Republic of China

E-mail: xin.wang2@siat.ac.cn

B. Fang

School of Chemistry and Materials Science

University of Science and Technology of China

Hefei, Anhui, 230026, People's Republic of China

Experimental Section

**Chemicals:** Ammonium molybdate [(NH_4_)_6_Mo_7_O_24_·4H_2_O] (99%), hydrochloric acid (HCl, 36.0-38.0%), Hydrazine monohydrate (N_2_H_4_·H_2_O, ≥50%) and hydrogen peroxide solution (H_2_O_2_, 30wt% in H_2_O) were purchased from Aladdin, Sodium chloride (NaCl, 99.5%), ethyl alcohol (C_2_H_5_OH, 99.7%), Iron(III) chloride hexahydrate (FeCl_3_·6H_2_O, 98%), Sodium acetate (CH_3_COONa, AR) and ammonium chloride (NH_4_Cl, 99.5%) obtained from Sinopharm Chemical Reagent Co., Ltd. Sodium citrate dehydrate (C_6_H_5_Na_3_O_7_·2H_2_O, 99%), sodium hydroxide (NaOH, 96%), hypochlorite solution (NaClO, 6-14%), sodium nitroferricyanidedihydrate (C_5_FeN_6_Na_2_O·2H_2_O, 99.98%), p-dimethylaminobenzaldehyde (C_9_H_11_NO, 99%) were provided by Macklin. Nafion solution and 117 Nafion membrane were provided by DuPont. All chemical reagents were used without further purification.

**Characterization:** The X-ray diffraction (XRD) was performed on a Bruker D8-Advance powder X-ray diffractometer with Cu-Kα radiation (λ = 1.5418 Å). Scanning electron microscopy (SEM) images were observed on a ZEISS scanning electron microscope (Gemini 300). Transmission electron microscopic (TEM) images were recorded by a JEOL/JEM-F200 electron microscope with an accelerating voltage of 200 kV. X-ray photoelectron spectroscopy (XPS) measurements were performed on a Thermo Fisher ESCALAB XI photoelectron spectrometer with Al Kα X-ray radiation as the X-ray source for excitation. The UV-Vis absorption spectra were recorded by HATACHI/UH4150 spectrometer. Nitrogen adsorption-desorption tests were conducted on Autosorb-iQC surface and porosity analyzer, and specific surface areas were calculated through the Brunaure-Emmert-Teller (BET) method. All electrochemical experiments were performed using CHI 760E electrochemistry workstation (CH Instruments, Inc., Shanghai).

**Synthesis of Fe_3_N-MoN, Fe_3_N and MoN:** The synthesis of Fe_3_N-MoN involves three steps. In the first step, 0.5 g FeCl_3_·6H_2_O and 1.9 g CH_3_COONa were dissolved in a mixed solution of 30 mL ethanol and 1.5 mL H_2_O with stirring for 30 min. Then the mixture was transferred into 50 mL Teflon autoclave and heated at 180 ℃ for 24 h. After cooling down to room temperature, the product was washed by deionized water and ethanol three times. The obtained product was α-Fe_2_O_3_. In the second step, 100 mg α-Fe_2_O_3_ and 55 mg (NH_4_)_6_Mo_7_O_24_·4H_2_O (Mo7) were dissolved into 40 mL ethanol. 5 g NaCl powder was added into the above precursor solutions. Then, the mixture solution was dried at 60 ℃ with continuous stirring. The obtained mixture was named α-Fe_2_O_3_/Mo7@NaCl. In the third step, the powder of α-Fe_2_O_3_/Mo7@NaCl was calcined at 600 ℃ under an NH_3_ atmosphere maintained for 3 h with a ramp rate of 2 ℃ min^-1^. After being cooled down naturally, the resultant product of Fe_3_N-MoN@NaCl was purified by repeatedly washing with deionized water to remove the NaCl salt templates. After washing, the final products of Fe_3_N-MoN were dried at 60 ℃ using a vacuum oven. The synthesis procedure of Fe_3_N and MoN is exactly the same as the procedure above, except that, no Mo7 is added into precursor solution for the preparation of Fe_3_N and no α-Fe_2_O_3_ is added into precursor solution for the preparation of MoN.

**Electrochemical measurements:** The NRR measurements were performed by an electrochemical station (CHI 760E) in a typical two-compartment H-type cell, separated by a Nafion 117 membrane. Before the test, the Nafion membrane was boiled in H_2_O_2_ (5%) aqueous solution and deionized water at 80 °C for 1h, respectively. The electrochemical experiments were conducted using a standard three-electrode system in an electrochemistry workstation, in which a Pt foil and a saturated Ag/AgCl electrode were acted as counter electrode and reference electrode, respectively. The working electrode was prepared as follows. The as-prepared catalyst was ultrasonically dispersed into a mixed solution consisting of 480 μL ethyl alcohol, 480 μL water and 40 μL 5 wt.% Nafion solution for at least 1 h. Then, the catalyst ink was dropped on a piece of carbon paper sized 1.0 cm × 1.0 cm, acted as the working electrode. The catalyst loading mass is 0.1 mg cm^-2^. All potentials were converted to RHE with the equation: E (vs. RHE) =E (vs. Ag/AgCl) + 0.059 × pH + 0.197. For NRR process, the potentiostatic tests were conducted in 40 mL N_2_ saturated 0.1 M HCl electrolyte, which was purged by feeding pure N_2_ gas into cathodic compartment for 0.5 h before measurement. The electrochemical active surface area (ECSA) was estimated by measuring the double-layer capacitance (C_dl_) associated with the CV cycles at the different scan rates from 120 mV s^-1^ to 200 mV s^-1^. The C_dl_ was obtained according to the following equation:

C_dl_ =$\frac{(Ja-Jc)/2}{v}$

in which Ja is the positive scan current at 0.15 V vs. RHE, Jc is the negative scan current at 0.15 V vs. RHE and v is the scan rate.

**Determination of ammonia:** The concentration of produced NH3 in the electrolyte was spectrophotometrically measured by the indophenol blue method. Typically, 2 mL of electrolyte was taken from the cathodic chamber, and then dispersed into 2 mL of 0.1 M NaOH solution consisting of salicylic acid (5%) and sodium citrate (5%), followed by the addition of 1.0 mL NaClO (0.05 M) and 0.2 mL C5FeN6Na2O (1 wt.%). After standing for 2 h, UV-Vis absorption spectrum at 655 nm was collected for further calculation of produced NH3. The concentration-absorbance calibration curve was created using standard NH_4_Cl (Figure. S8).

**Determination of N_2_H_4_:** The concentration of possible byproduct N2H4 was measured by the Watt and Chrisp method. In detail, a mixture of 5.99 g p-dimethylaminobenzaldehyde, 30 mL concentrated HCl and 300 mL ethanol was acted as a color reagent, followed by reacting with 5 mL electrolyte after electrolysis. The mixed solution was stirred for 20 min at room temperature, then UV-Vis absorption spectrum of the resulting solution was recorded at 455 nm. The concentration-absorbance calibration curve was built using standard N_2_H_4_·H_2_O (Figure. S9).

**Determination** **of NH_3_ yield and FE:** The NH3 yield rate was calculated using the following equation:

Yield rate (NH_3_) = (C (NH_3_)×V)/(m (cat.)×t)

where C(NH3) is the concentration of measured NH3, V is the volume of the 0.1M HCl electrolyte, t is the reduction reaction time, and m is the loading mass of catalyst.

The Faradaic efficiency (FE) was calculated using the following equation:

FE = (3×F×C (NH_3_) ×V)/(17 × Q) ×100%

where F is the Faraday constant, C (NH3) is the concentration of measured NH3, V is the volume of the 0.1 M HCl electrolyte, Q is the charge accumulated for 1 h electrocatalysis.

**^15^N Isotopic Labeling Experiment:** ^15^N isotopic labeling experiment was carried out using ^15^N_2_ (Sigma-Aldrich, 99%) as supply gas. Prior to electrolysis, using ultrapure argon (99.999%) gas to continuously bubble into the 0.1 M HCl solution for 30 minutes to remove residual ^14^N_2_. In the process of NRR electrolysis, the ^15^N_2_ gas continuously bubble into the electrolyte and electrolyzed at -0.2 V *vs* RHE for 1h. Then, the produced ^15^NH_4_^+^ was detected through ^1^H nuclear magnetic resonance (NMR) spectra. Detailedly, 10 ml of the reaction solution was removed from the cathodic chamber and concentrated to 1 mL, and then the pH was adjusted to 2. Then take 30 μL of the above solution and dissolve it in 600 μL dimethyl sulfoxide-D6, followed by the measurement of ^1^H NMR spectra. ^14^N_2_ labeling experiment and the corresponding ^1^H NMR measurement were implemented using the same method. (^14^NH_4_)_2_SO_4_ and (^15^NH_4_)_2_SO_4_ solution were used as the standard substances of ^14^NH_4_^+^ and ^15^NH_4_^+^.

**DFT computation details:** All of the density functional theory (DFT) calculations were carried out by using Vienna ab-initio Simulation Package (VASP).^[1]^ The exchange-correlation energy was described with Perdew-Burke-Ernzerhof (PBE) functional with the generalized gradient approximation (GGA).^[2]^ DFT-D3 method was employed to calculate the van der Waals (vdW) interaction. The effect of core electrons on the valence electron density were treated by using Projector Augmented Wave (PAW) method. The cutoff energy for the plane wave basis sets was set to 480 eV. Electronic energies were computed with the tolerance of 1 × 10^-4^ eV and total force of 0.02 eV/Å. The Brillouin zone was sampled by k-points mesh of 3 × 3 × 1. A vacuum space of 15 Å was inserted in z direction to avoid interactions between periodic images.

The Gibbs free energy change (ΔG) of N_2_ reduction reaction steps was determined as follows:

∆G = ∆E + ∆ZPE - T∆S

where ΔE is the electronic energy difference directly obtained from DFT calculations, ΔZPE is the zero-point energy difference, T is the room temperature (298.15 K) and ΔS is the entropy change.^[3]^ The entropies of free gases are taken from standard values.


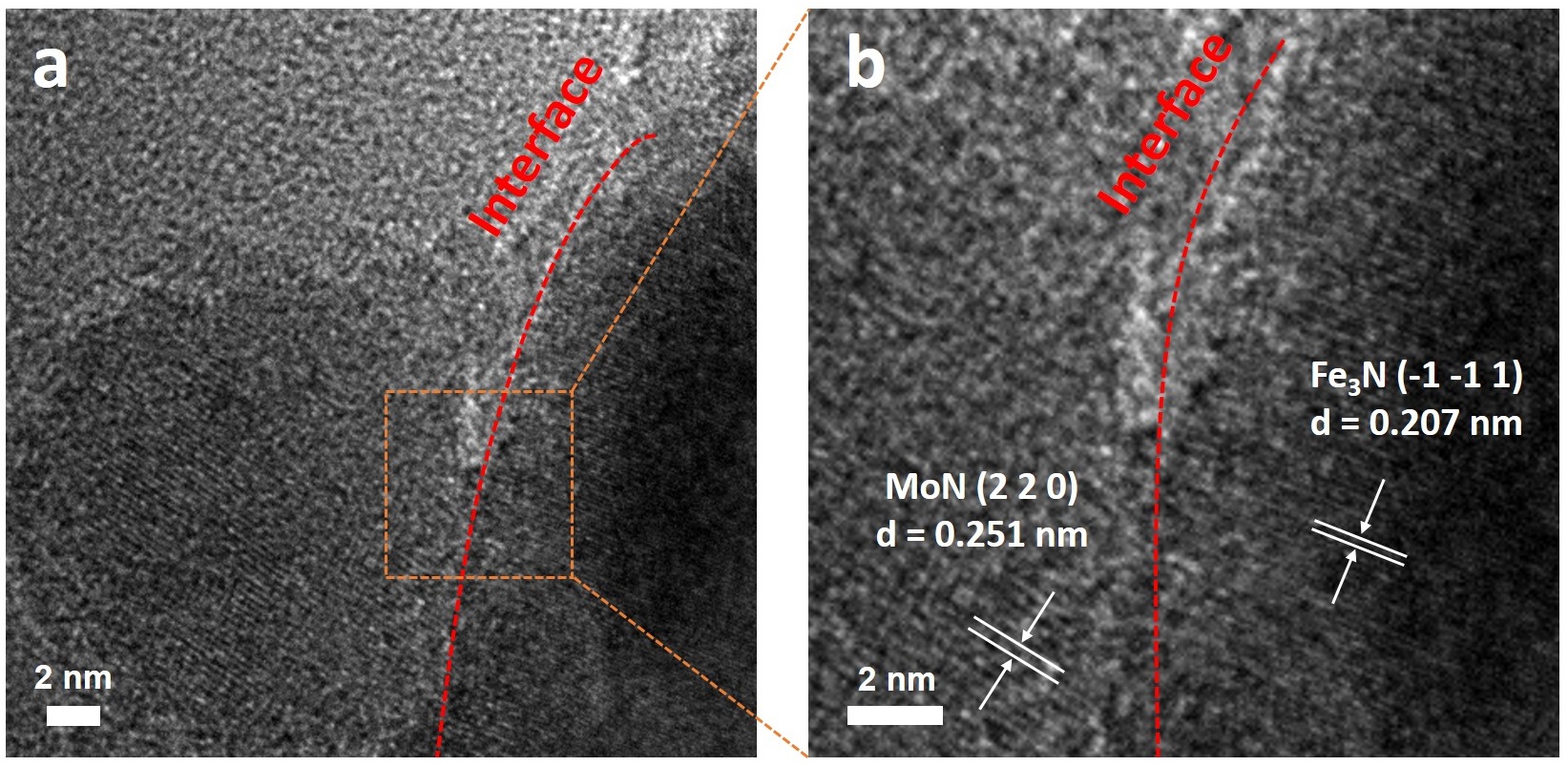


**Figure S1.** HRTEM images of Fe_3_N-MoN.


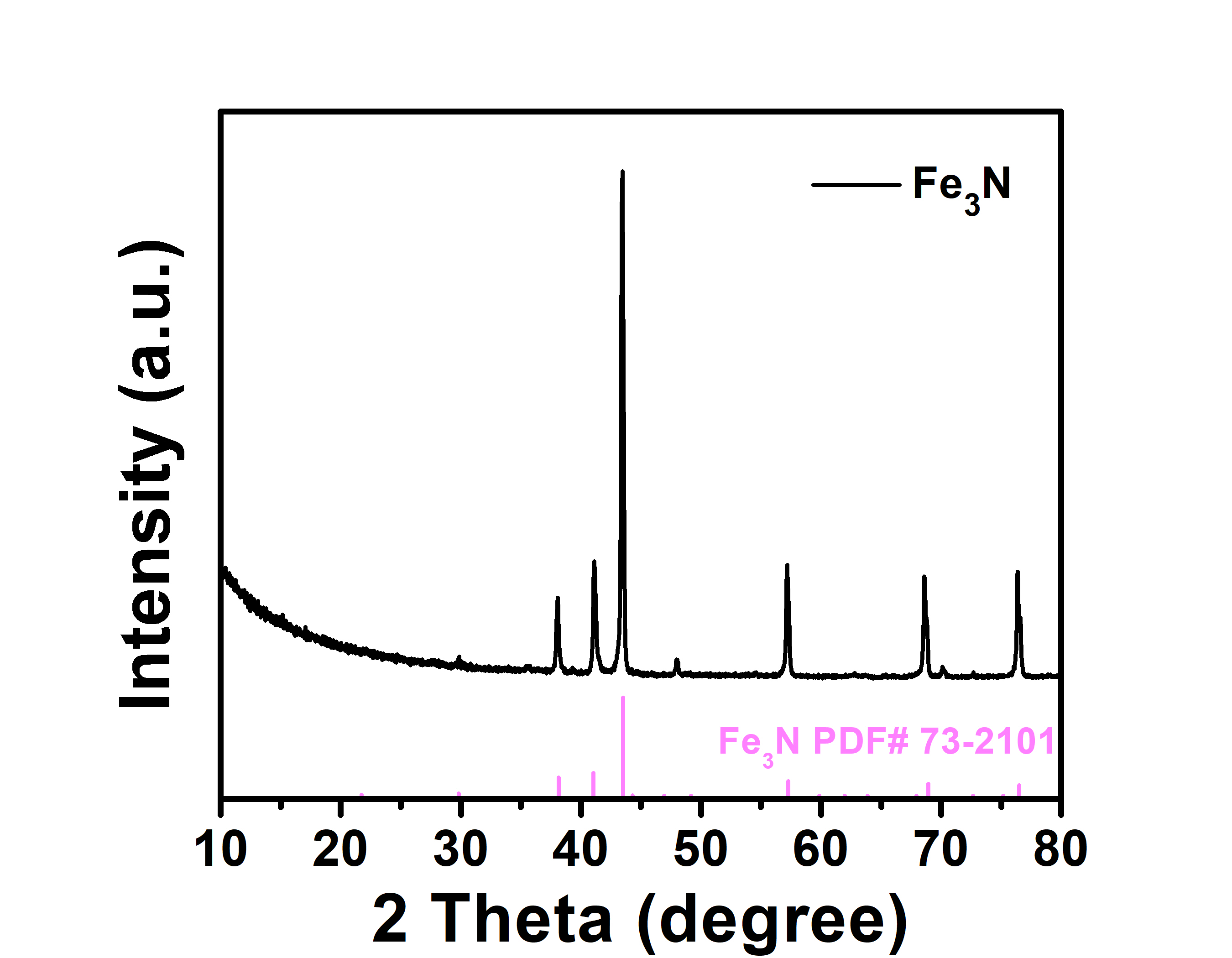


**Figure S2.** XRD pattern of the Fe_3_N.


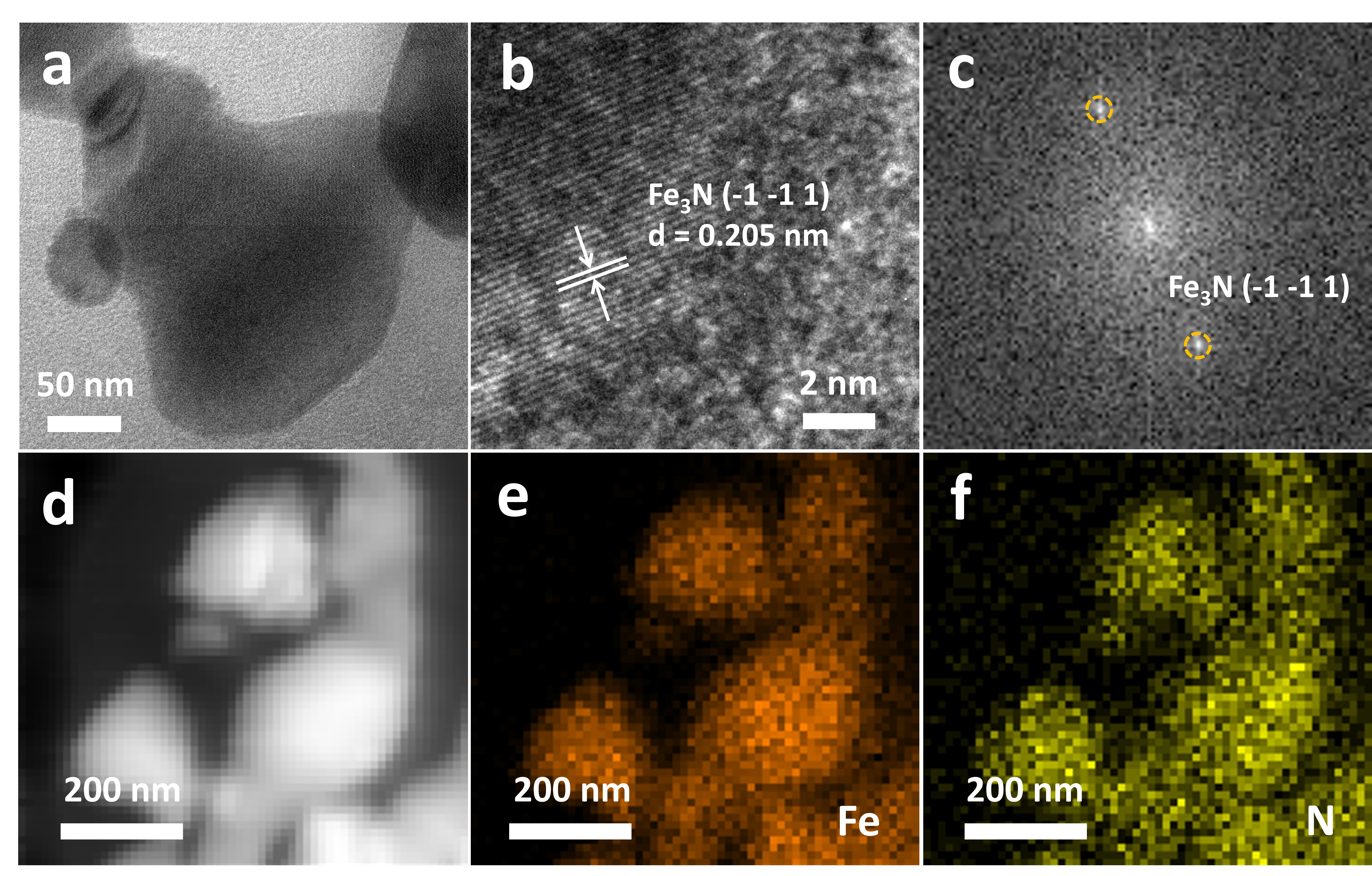


**Figure S3.** (**a**) TEM image of Fe_3_N. (**b**) HRTEM image of Fe_3_N. (**c**) The selected area FFT pattern from **b**. (**d-f**) HAADF-STEM images with corresponding element mapping analyses of Fe_3_N.


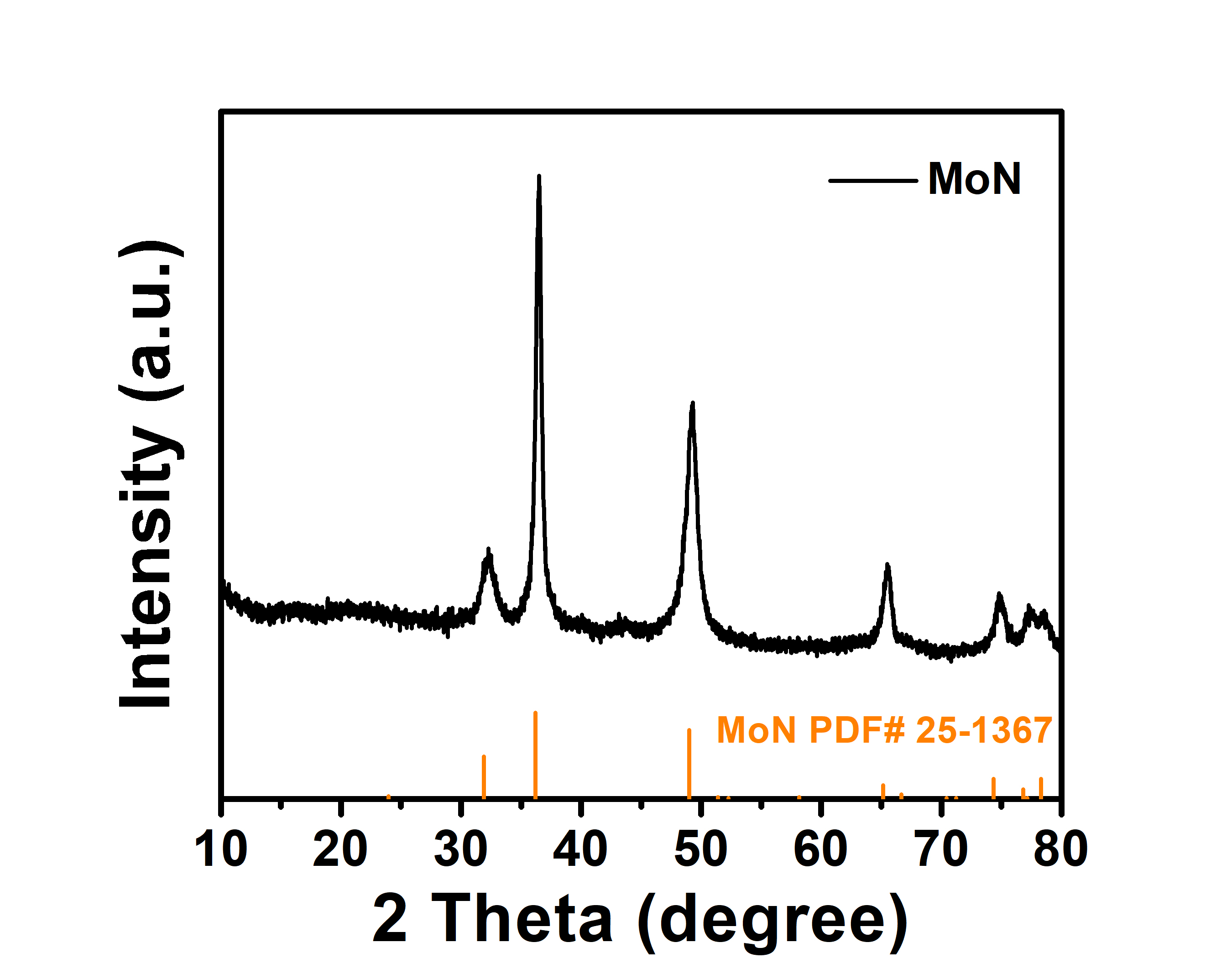


**Figure S4.** XRD pattern of the MoN.


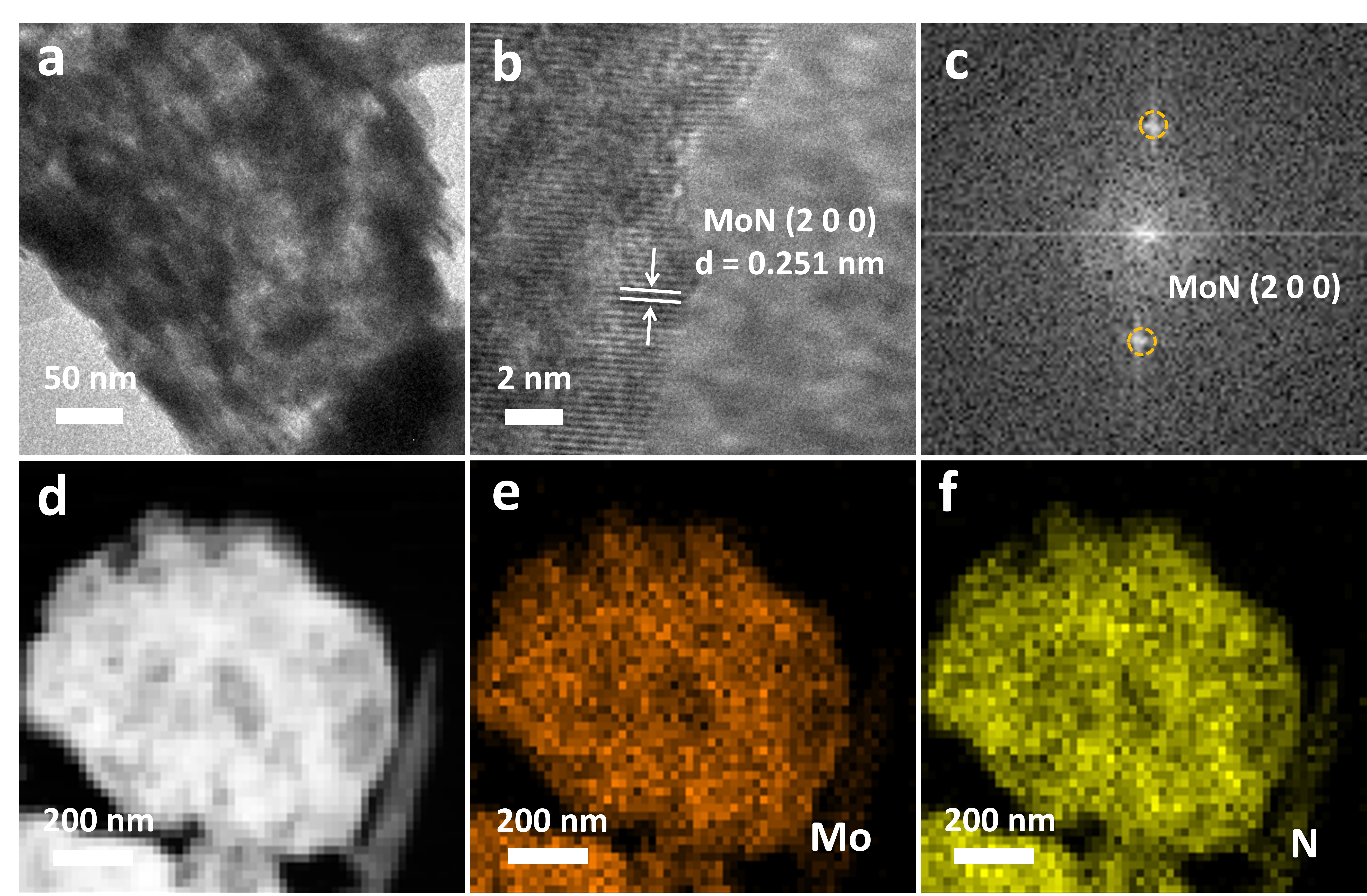


**Figure S5.** (**a**) TEM image of MoN. (**b**) HRTEM image of MoN. (**c**) The selected area FFT pattern from **b**. (**d-f**) HAADF-STEM images with corresponding element mapping analyses of MoN.


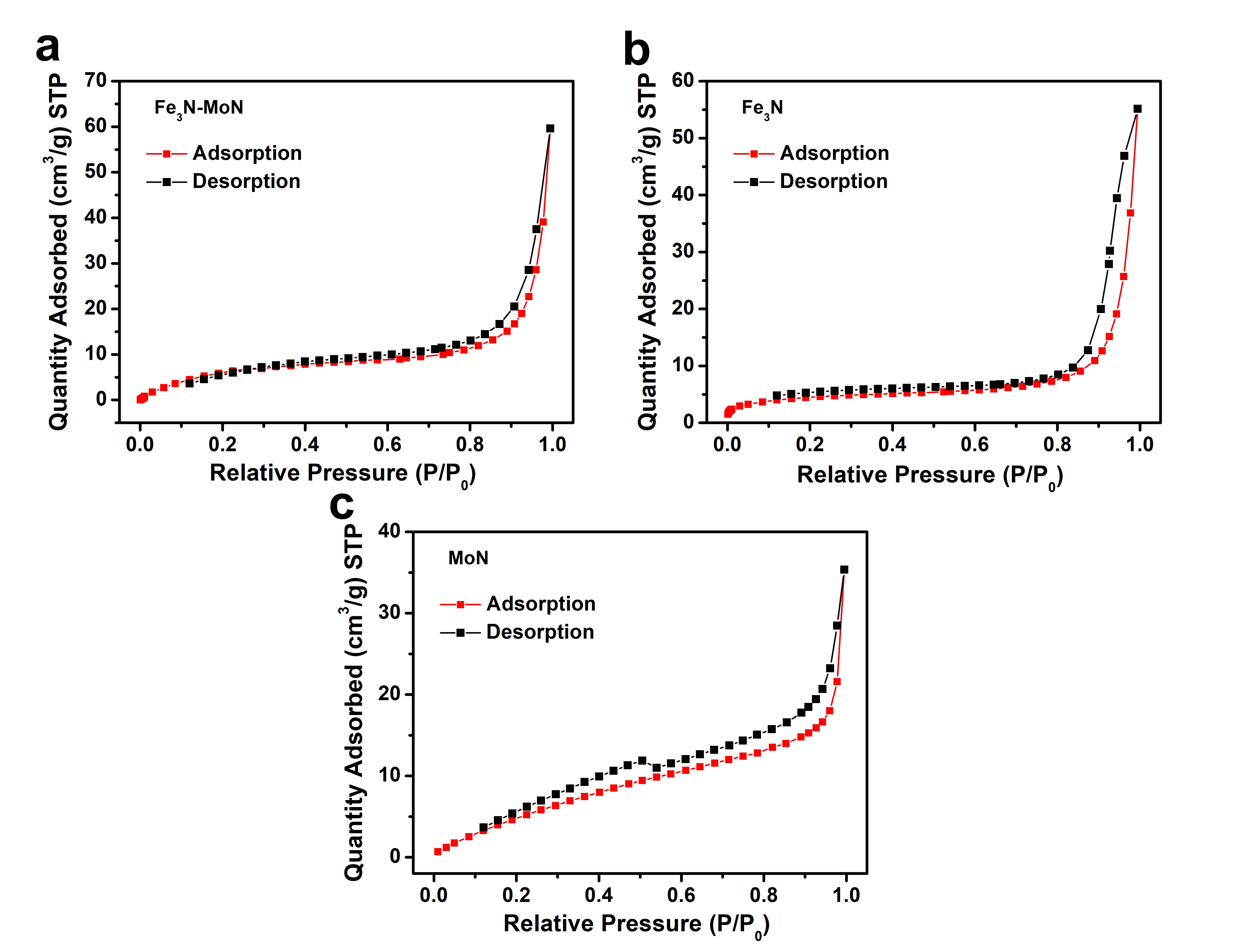


**Figure S6.** Nitrogen adsorption-desorption isotherms of (**a**) Fe_3_N-MoN, (**b**) Fe_3_N, and (**c**) MoN.


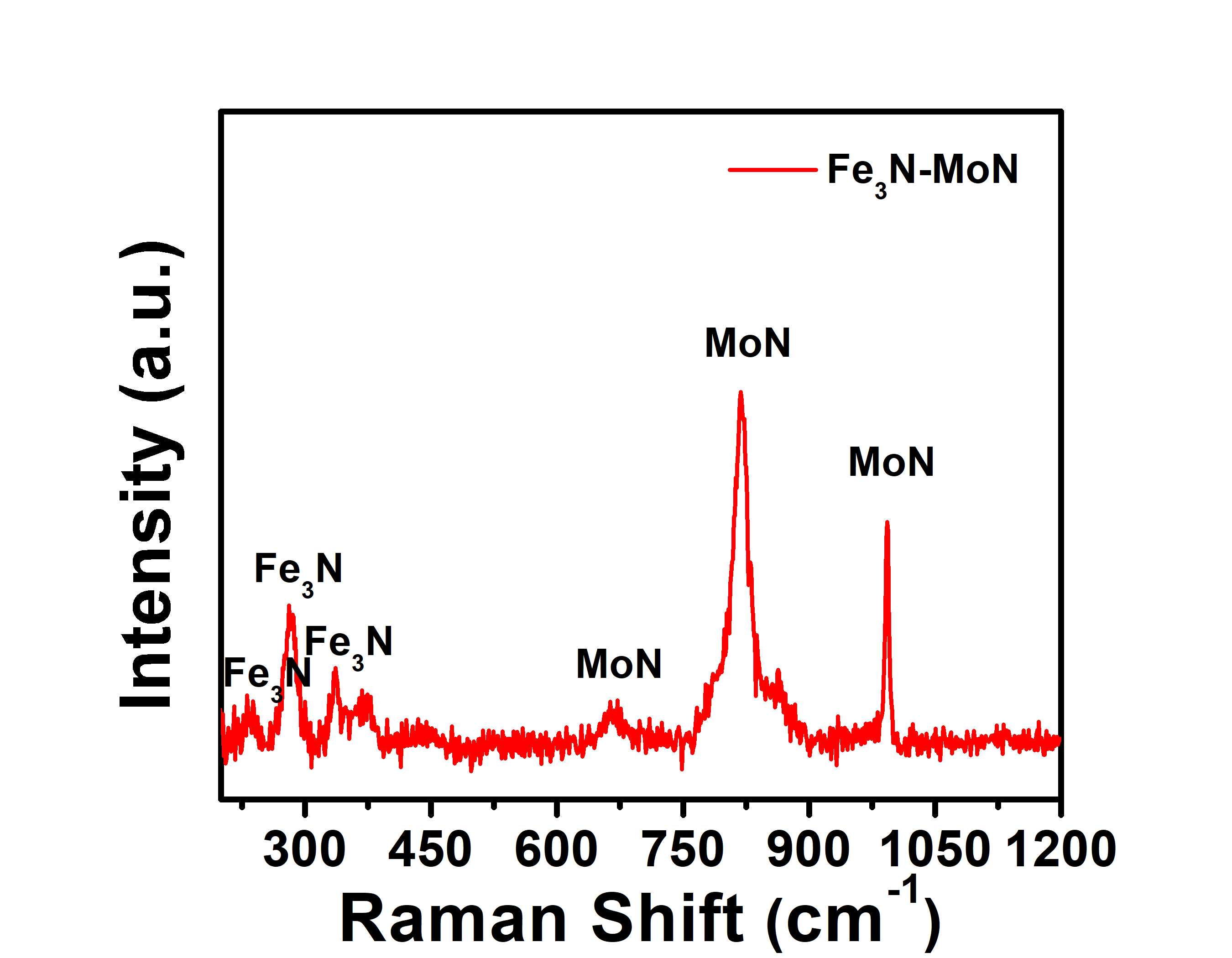


**Figure S7.** Raman spectrum of the Fe_3_N-MoN.


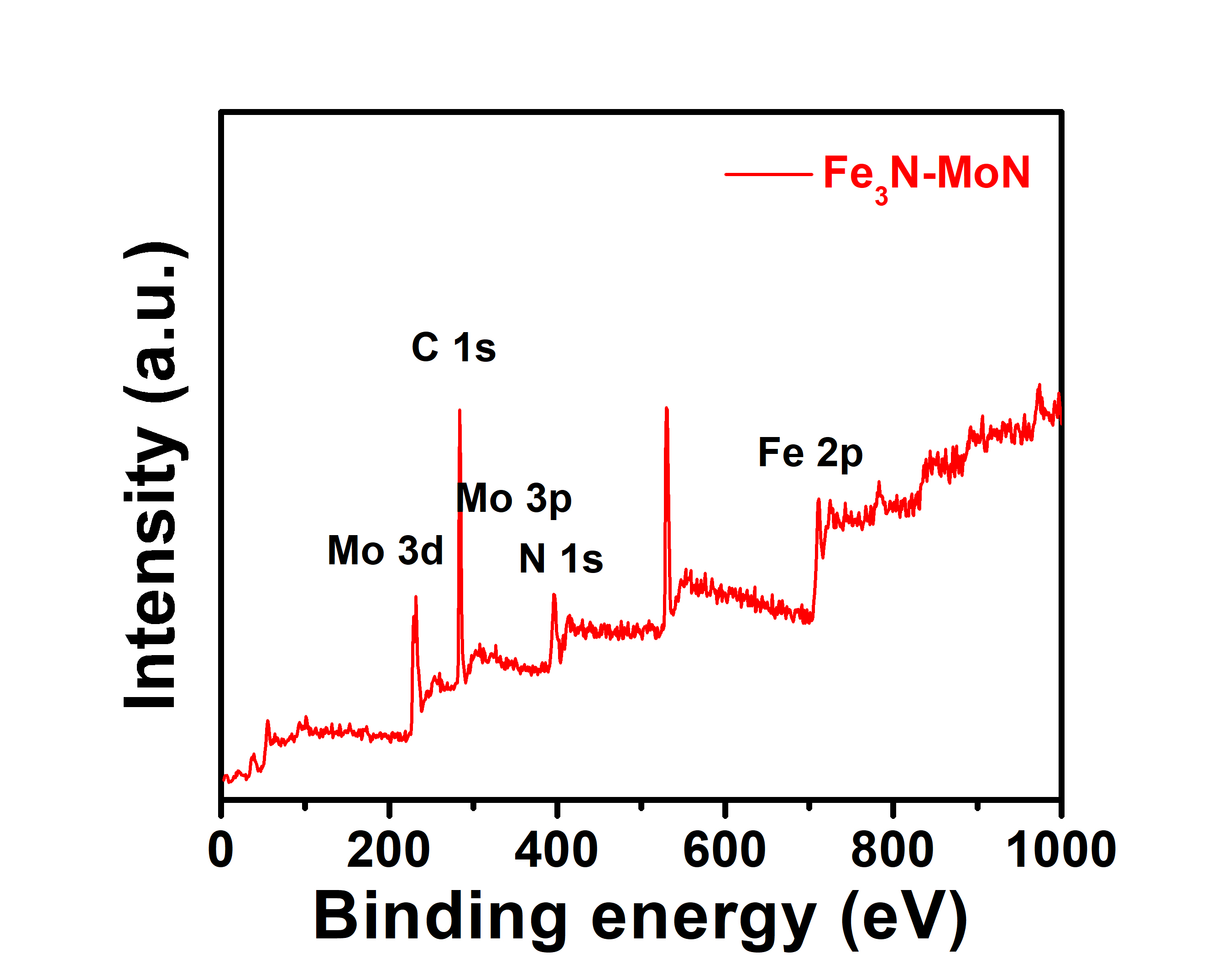


**Figure S8.** XPS survey spectrum of the Fe_3_N-MoN.


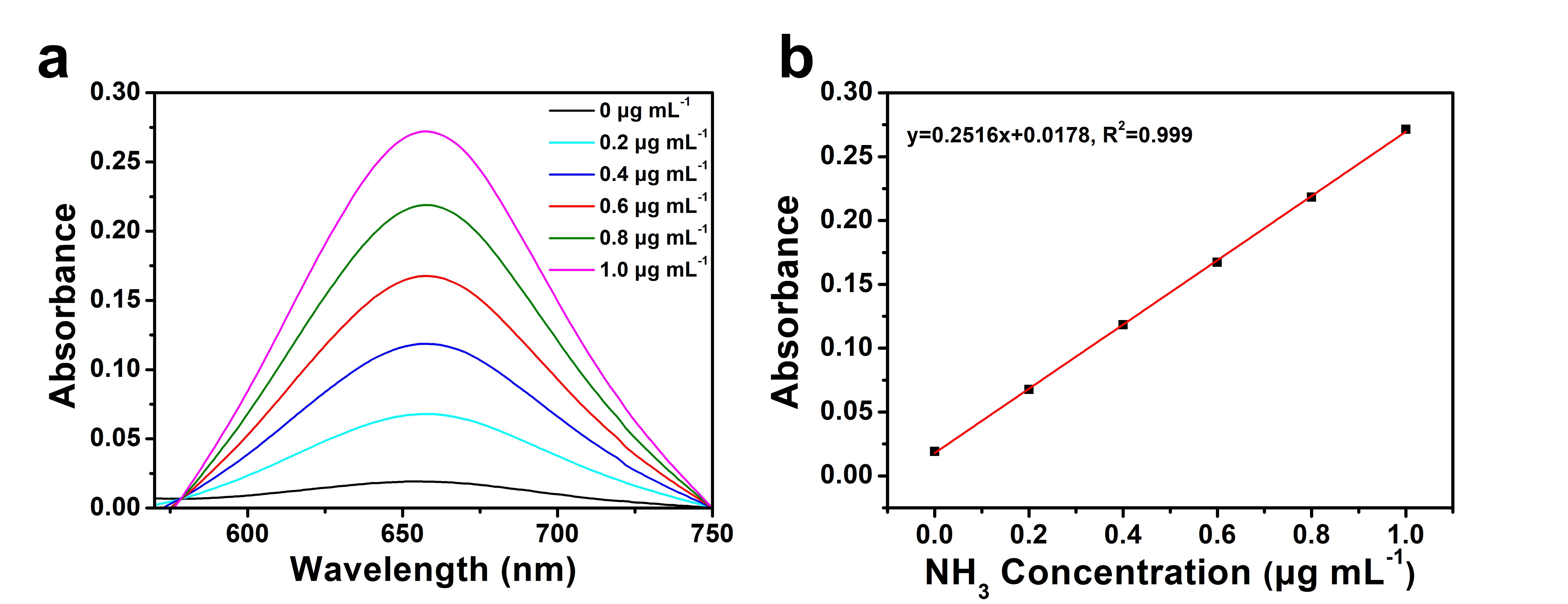


**Figure S9.** (**a**) UV-Vis absorption spectra of various NH_3_ concentrations after incubated for 2h at room temperature. (**b**) Calibration curve used for calculation of NH_3_ concentrations.


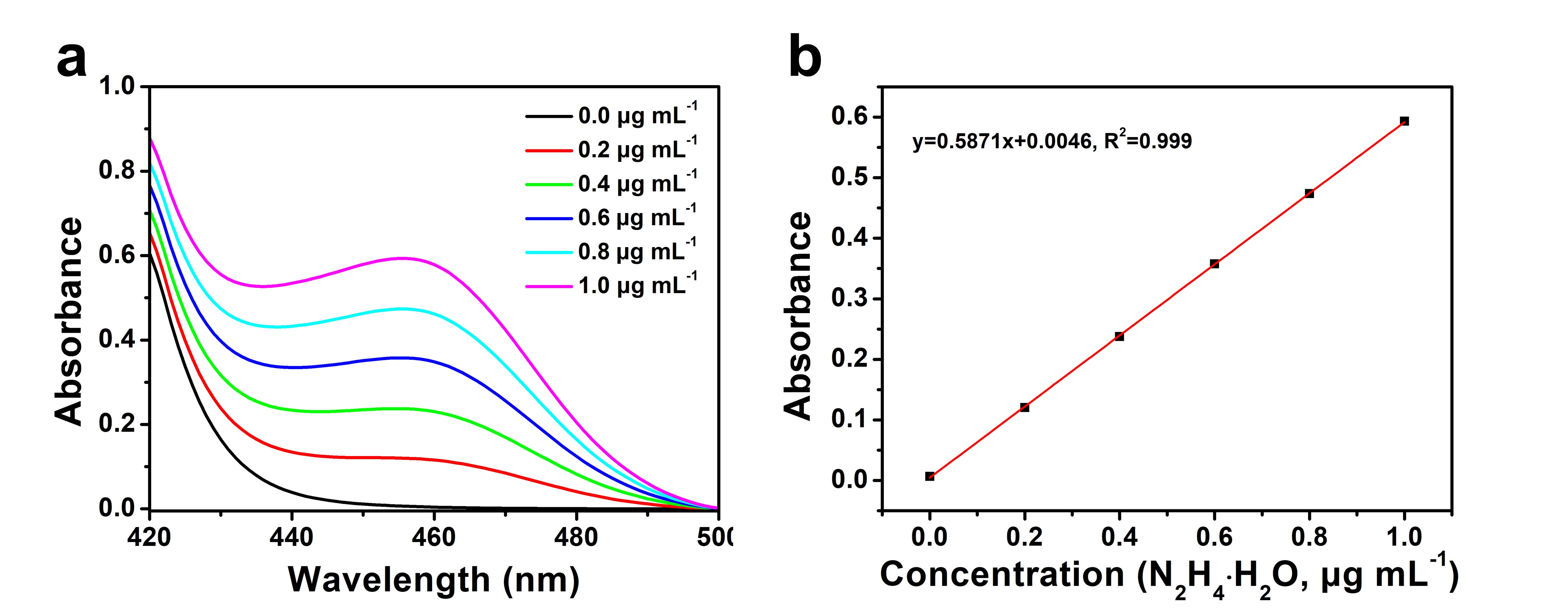


**Figure S10.** (**a**) UV-Vis absorption spectra of various N_2_H_4_·H_2_O concentrations after incubated for 10 min at room temperature. (**b**) Calibration curve used for calculation of N_2_H_4_·H_2_O concentrations.


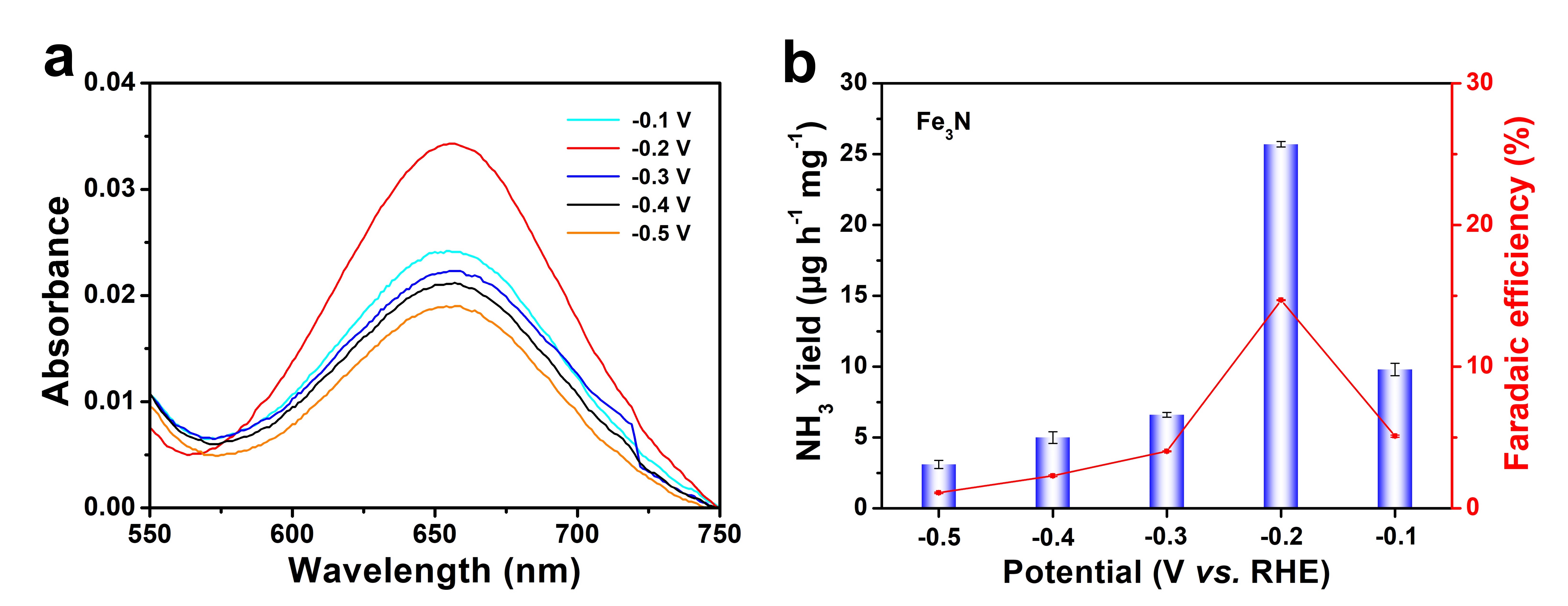


**Figure S11.** (**a**) UV-vis absorption spectra of electrolyte stained with indophenol indicator for Fe_3_N at different potentials. (**b**) Corresponding calculated NH_3_ yield and FEs at each given potentials.


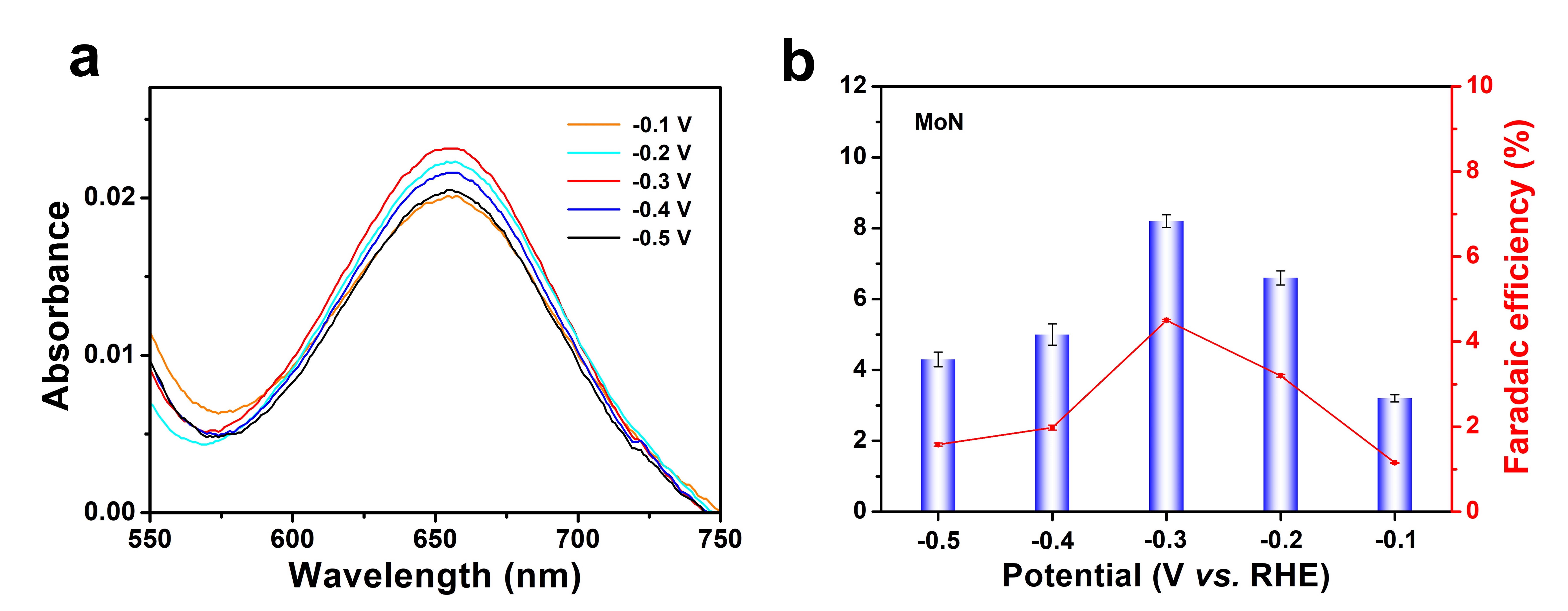


**Figure S12.** (**a**) UV-vis absorption spectra of electrolyte stained with indophenol indicator for MoN at different potentials. (**b**) Corresponding calculated NH_3_ yield and FEs at each given potentials.


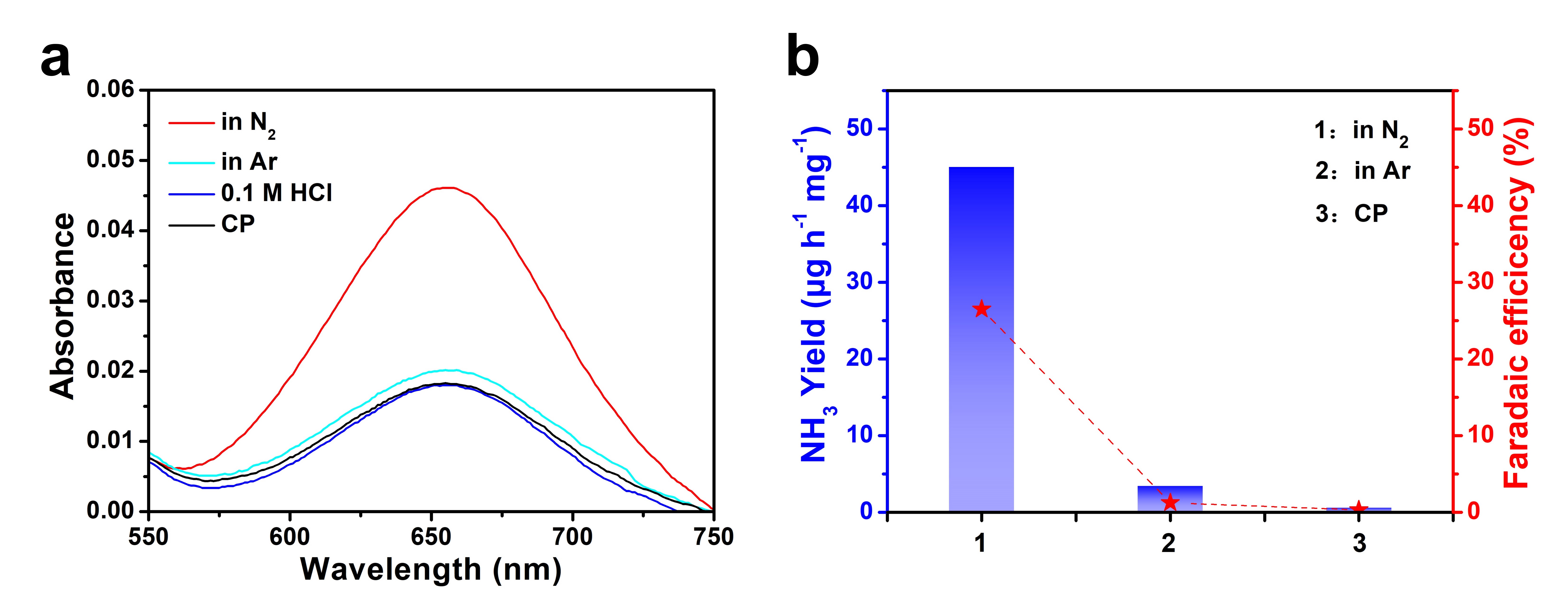


**Figure S13.** (**a**) UV-vis absorption spectra of electrolyte stained with indophenol indicator for 2 h under different conditions. (**b**) Corresponding calculated NH_3_ yield rates and FEs at a potential of -0.2 V for different electrodes.

**
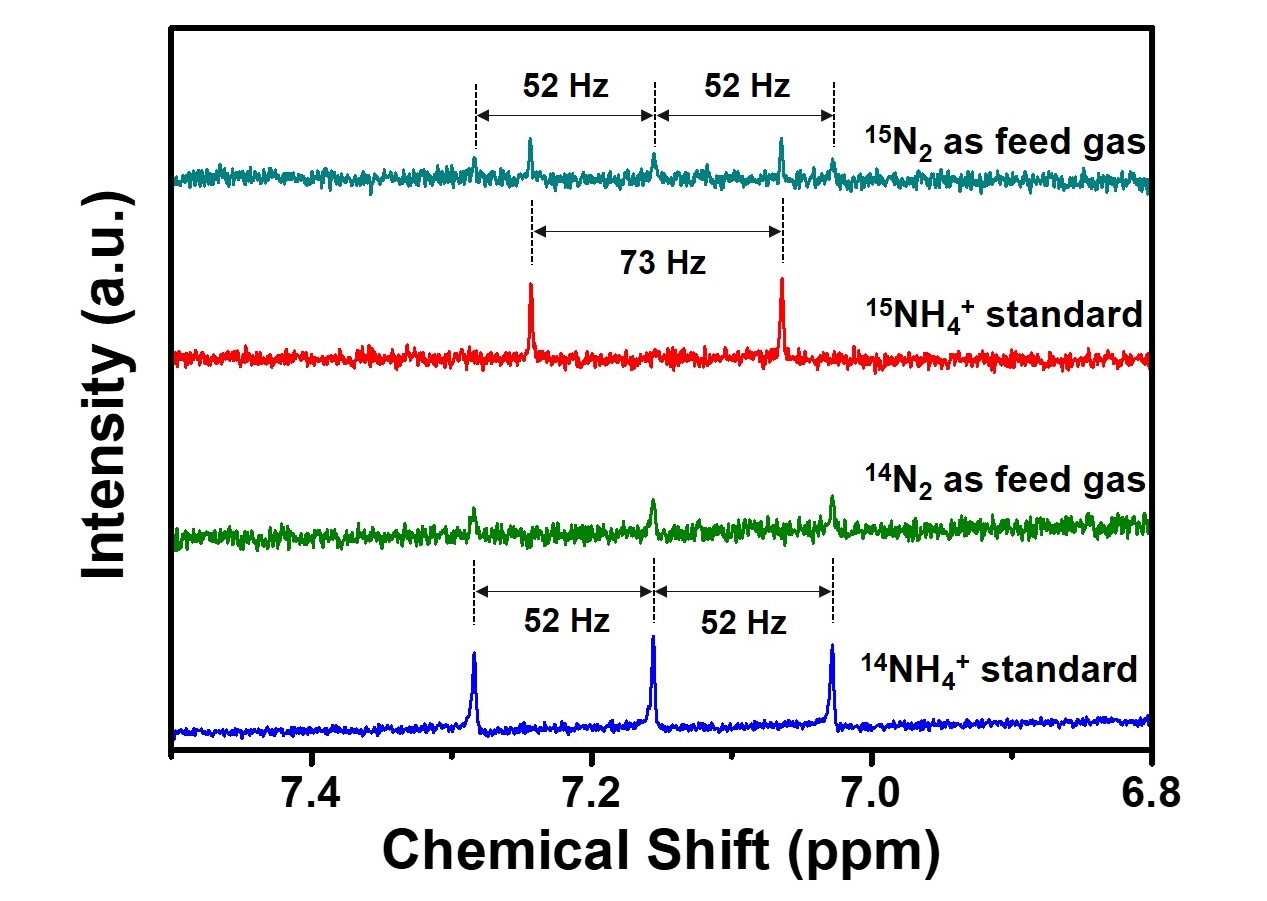
**

**Figure S14.** ^1^H nuclear magnetic resonance (NMR) spectra of the ^14^NH_4_^+^ and ^15^NH_4_^+^ standard samples and the electrolyte fed by ^14^N_2_ and ^15^N_2_ after NRR.


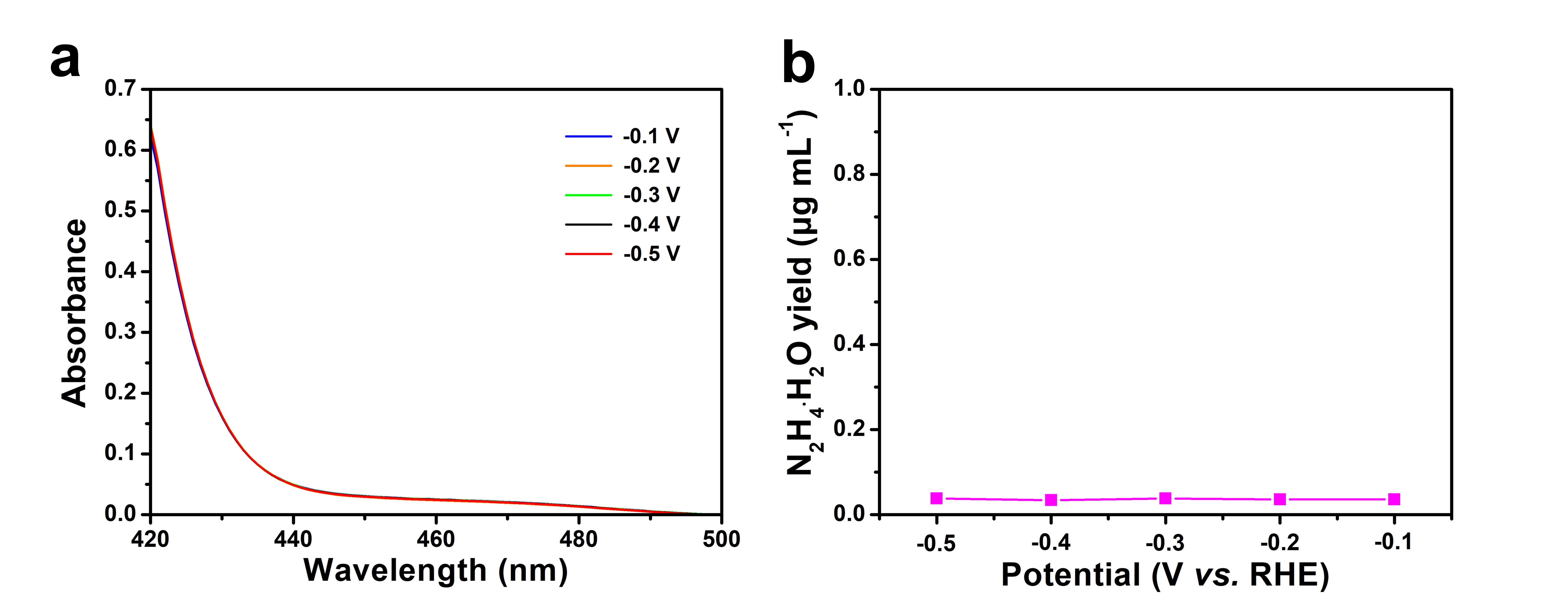


**Figure S15.** (**a**) UV-Vis spectra of the electrolyte estimated by the method of Watt and Chrisp after NRR process for Fe_3_N-MoN. (**b**) Corresponding calculated N_2_H_4_·H_2_O concentration.

**
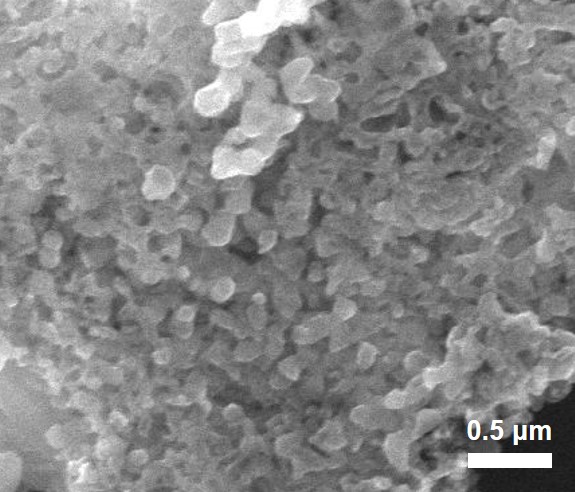
**

**Figure S16.** SEM image of Fe_3_N-MoN after the stability test.


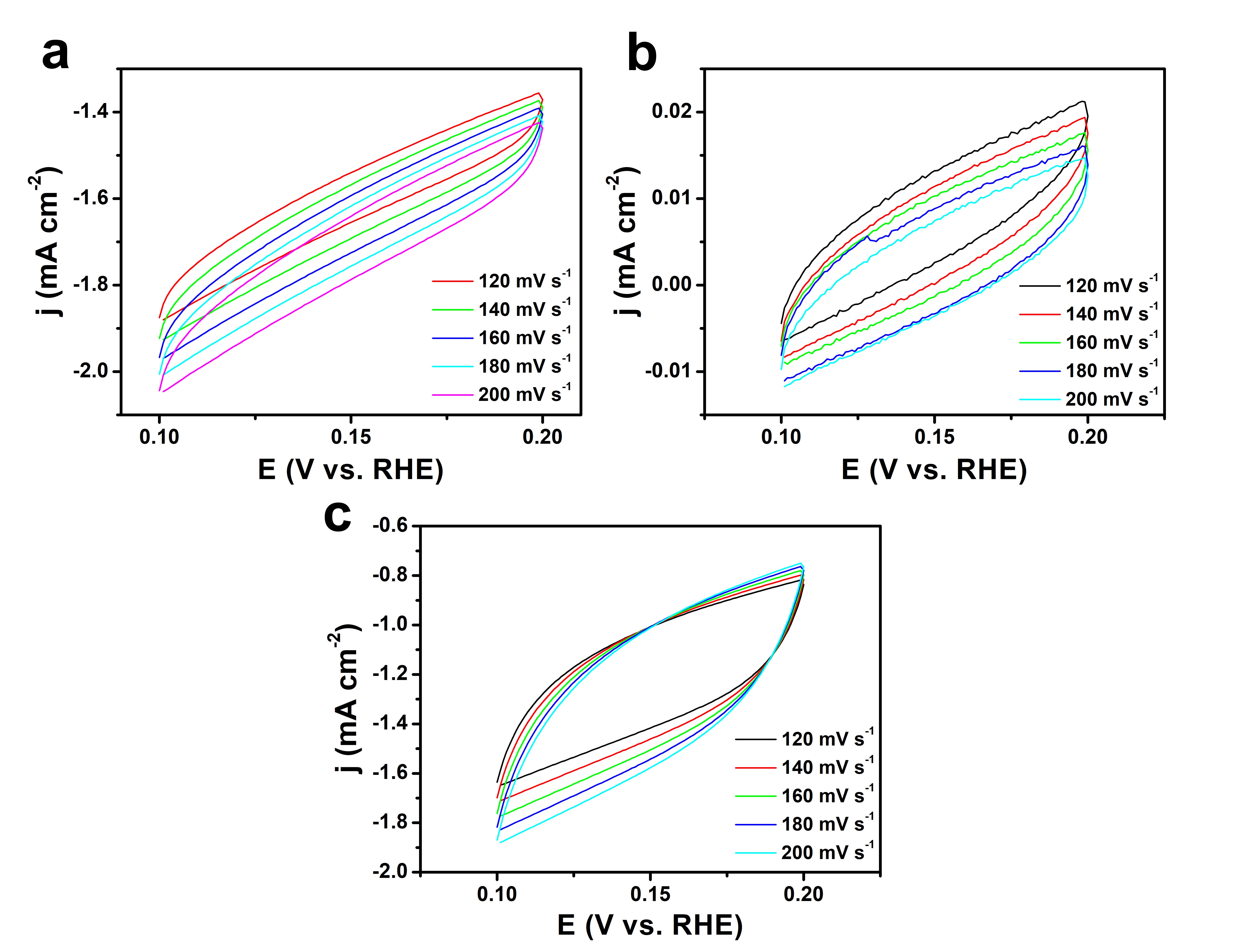


**Figure S17.** CV curves of (**a**) Fe_3_N-MoN, (**b**) Fe_3_N and (**c**) MoN at 120-200 mV s^-1^ in the range of 0.1 and 0.2 V vs. RHE.


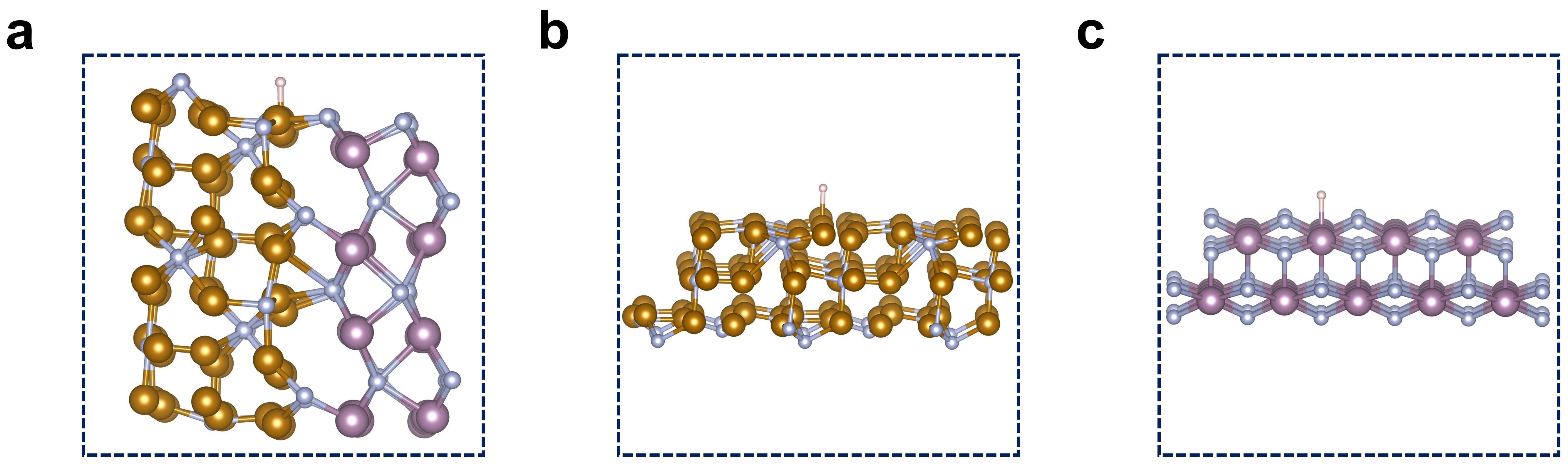


**Figure S18.** Atomistic structure of H adsorbed (**a**) Fe_3_N-MoN, (**b**) Fe_3_N and (**c**) MoN.





**Figure S19.** The schematic diagrams of NRR process over Fe_3_N-MoN via the (**a**) distal pathway, (**b**) alternating pathway and (**c**) MvK pathway.


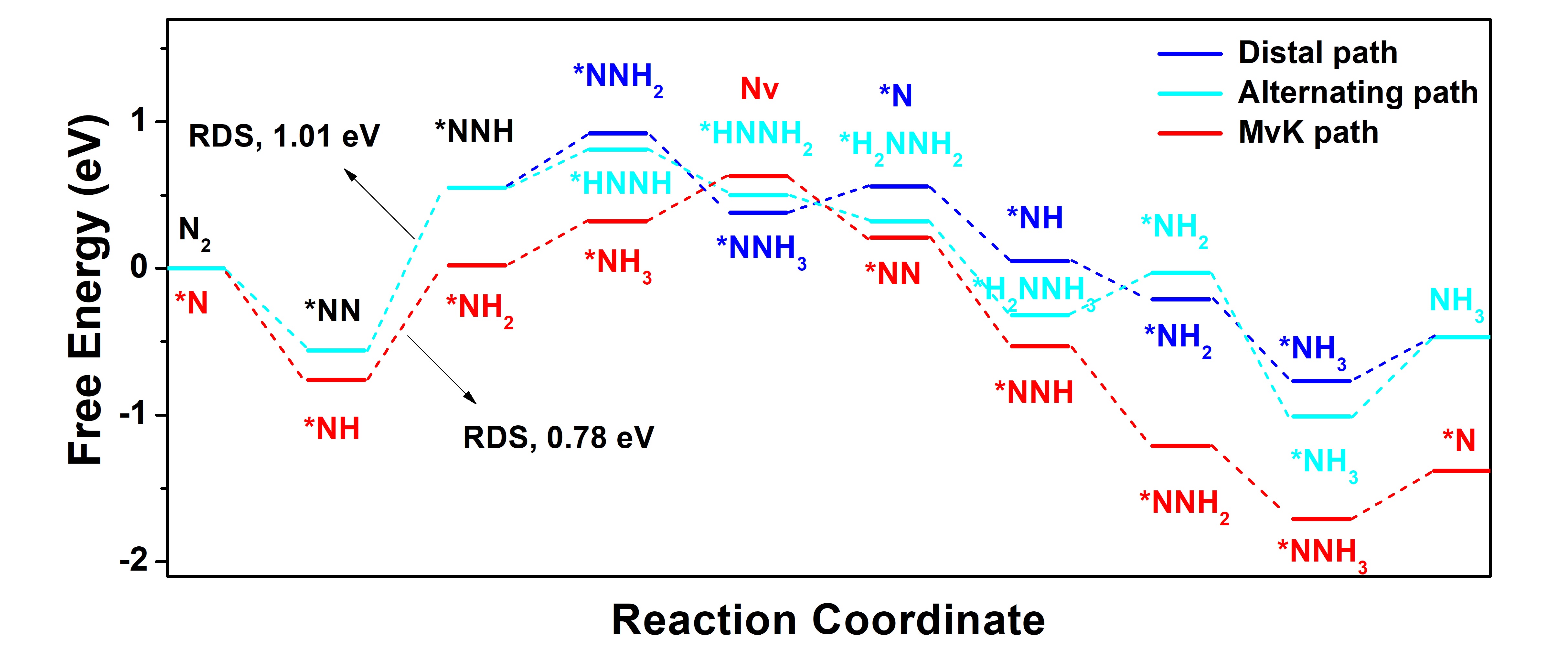


**Figure S20.** Gibbs free energy (ΔG) diagrams of NRR over Fe_3_N-MoN via the distal pathway, alternating pathway and MvK pathway.


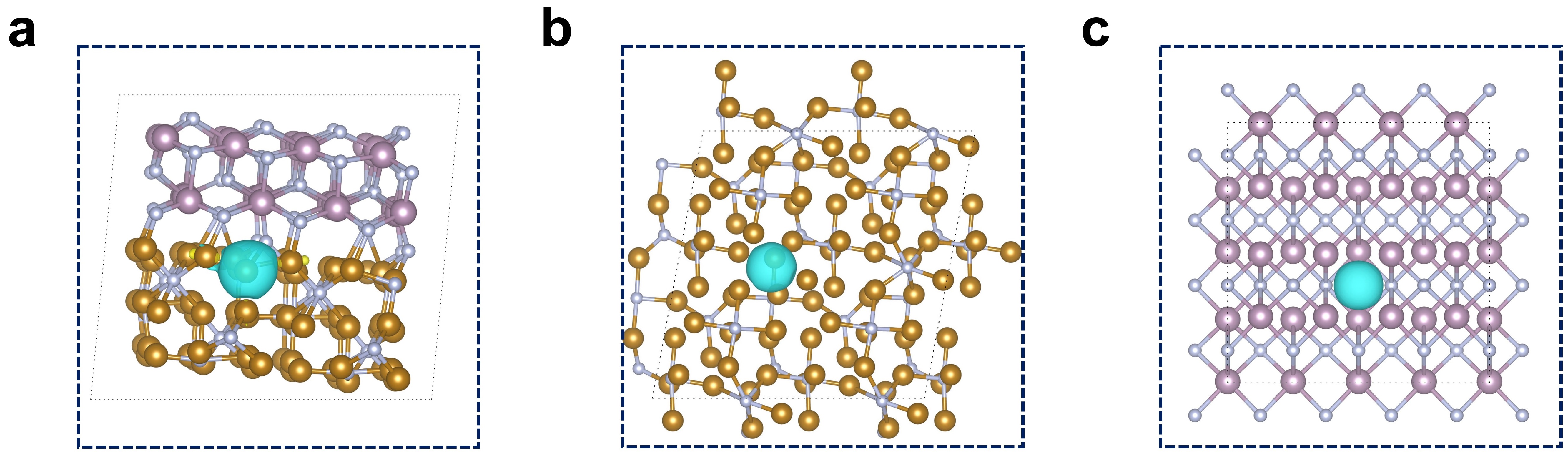


**Figure S21.** Charge density differences of the (**a**) Fe_3_N-MoN-Nv, (**b**) Fe_3_N-Nv and (**c**) MoN-Nv. The yellow and cyan electron clouds show accumulation and depletion of electrons, respectively. The isosurface value is 0.03 e Bohr^-3^.

**Reference**

1. G. G. Kresse, J.J. Furthmüller*, Phys. Rev. B* 1996, *54*, 11169.

2. P. E. Blöchl, Physical Review B 1994, 50, 17953-17979.

3. J. K. Nørskov, J. Rossmeisl, A. Logadottir, L. Lindqvist, J. R. Kitchin, T. Bligaard, H. Jónsson, The Journal of Physical Chemistry B 2004, 108, 17886-17892.
